# Coordinated labio-lingual asymmetries in dental and bone development create a symmetrical acrodont dentition

**DOI:** 10.1038/s41598-020-78939-2

**Published:** 2020-12-16

**Authors:** M. Kavková, M. Šulcová, J. Dumková, O. Zahradníček, J. Kaiser, A. S. Tucker, T. Zikmund, M. Buchtová

**Affiliations:** 1 Central European Institute of Technology, Brno University of Technology, Brno, Czech Republic; 2 Department of Experimental Biology, Faculty of Science, Masaryk University, Brno, Czech Republic; 3 Department of Histology and Embryology, Faculty of Medicine, Masaryk University, Brno, Czech Republic; 4 Department of Radiation Dosimetry, Nuclear Physics Institute, Czech Academy of Sciences, Prague, Czech Republic; 5 Centre for Craniofacial and Regenerative Biology, King’s College London, Floor 27 Guy’s Tower, Guy’s Hospital, London Bridge, London, UK; 6 Laboratory of Molecular Morphogenesis, Institute of Animal Physiology and Genetics, Czech Academy of Sciences, Veveri 97, 602 00 Brno, Czech Republic

**Keywords:** Body patterning, Bone development

## Abstract

Organs throughout the body develop both asymmetrically and symmetrically. Here, we assess how symmetrical teeth in reptiles can be created from asymmetrical tooth germs. Teeth of lepidosaurian reptiles are mostly anchored to the jaw bones by pleurodont ankylosis, where the tooth is held in place on the labial side only. Pleurodont teeth are characterized by significantly asymmetrical development of the labial and lingual sides of the cervical loop, which later leads to uneven deposition of hard tissue. On the other hand, acrodont teeth found in lizards of the Acrodonta clade (i.e. agamas, chameleons) are symmetrically ankylosed to the jaw bone. Here, we have focused on the formation of the symmetrical acrodont dentition of the veiled chameleon (*Chamaeleo calyptratus*). Intriguingly, our results revealed distinct asymmetries in morphology of the labial and lingual sides of the cervical loop during early developmental stages, both at the gross and ultrastructural level, with specific patterns of cell proliferation and stem cell marker expression. Asymmetrical expression of ST14 was also observed, with a positive domain on the lingual side of the cervical loop overlapping with the SOX2 domain. In contrast, micro-CT analysis of hard tissues revealed that deposition of dentin and enamel was largely symmetrical at the mineralization stage, highlighting the difference between cervical loop morphology during early development and differentiation of odontoblasts throughout later odontogenesis. In conclusion, the early asymmetrical development of the enamel organ seems to be a plesiomorphic character for all squamate reptiles, while symmetrical and precisely orchestrated deposition of hard tissue during tooth formation in acrodont dentitions probably represents a novelty in the Acrodonta clade.

## Introduction

**Figure 1:**
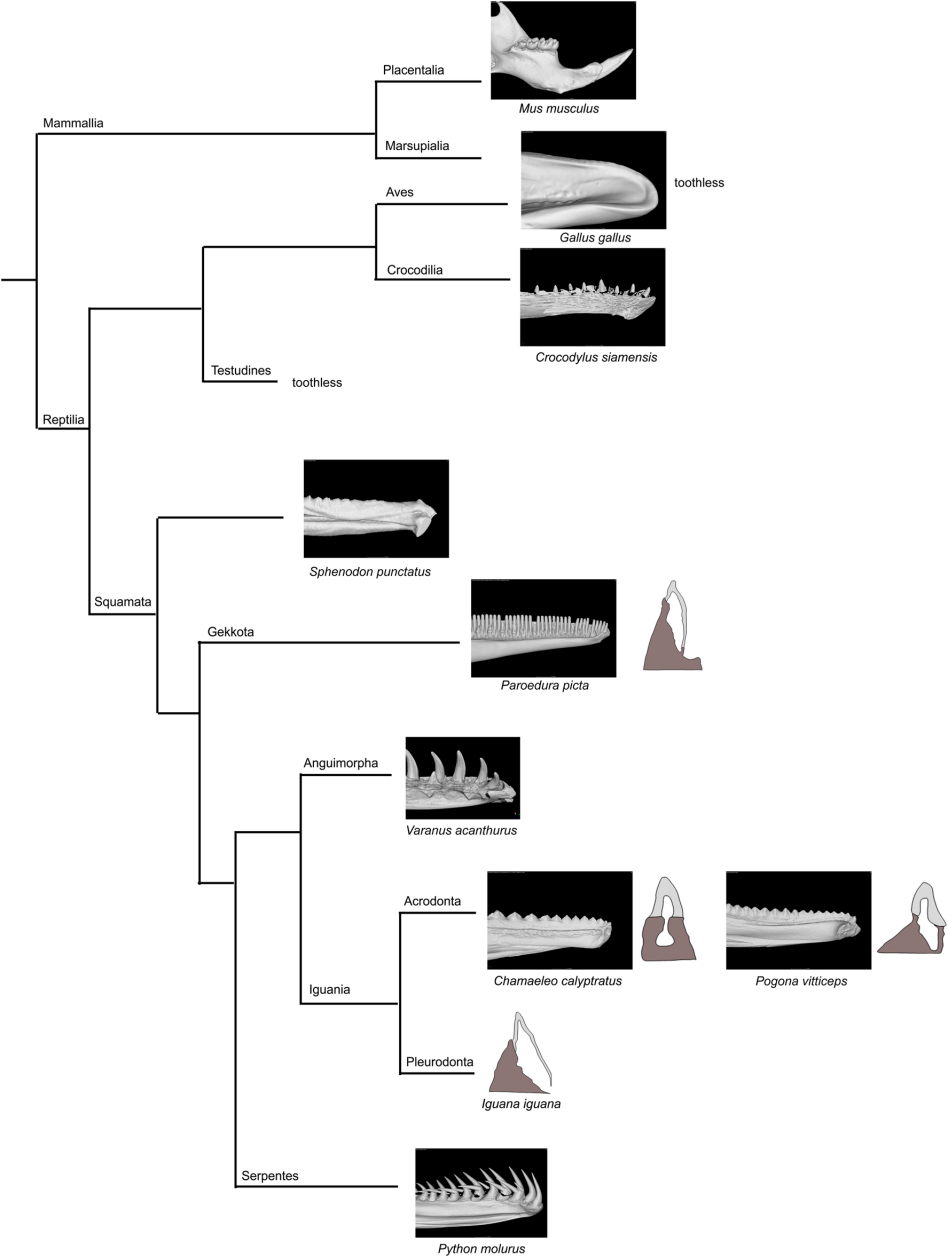
Simplified phylogenetic tree with displayed type of dentitions for selected species. Segment of the lower jaw in micro-CT view is displayed for selected vertebrate species to reveal possible tooth and bone relationship. Drawings of the transversal sections were modified based on^3^. Phylogenetic tree was adapted from^15^ and^16^.

Teeth are firmly anchored to the jaw and palatal bones in lepidosaurian reptiles (Squamata and Rhynchocephalia). Several modes of tooth-bone attachment evolved in this reptilian lineage (Fig. 1)^1–4^. In the majority of lizards and snakes, teeth are ankylosed to the inner side of the high labial wall of the tooth bearing element using a so called pleurodont implantation^5–7^. This type of attachment creates a space for the successional dental lamina growth and continuous tooth replacement (polyphyodonty) on the unattached lingual side of the tooth^8^. Enamel organs of tooth germs in species with pleurodontly ankylosed teeth demonstrate labio-lingual asymmetrical growth of the cervical loop from early stages of tooth development. As was previously described in the ocelot gecko (*Paroedura picta*), the lingual side of the cervical loop protrudes deep into the underlying mesenchyme, whereas the labial side of the tooth base is shorter, meets the bony pedicles and undergoes ankylosis (Fig. 1)^5^. This pleurodont mode of attachment has been proposed to be the plesiomorphic condition in Lepidosauria^3,9^.

In contrast, all teeth of extant chameleons and the majority of teeth in agamas are fused to the crest of the tooth-bearing skeletal element by acrodont ankylosis^3^. In this specific type of attachment, the tooth is located at the top of a bony pedicle. Once the tooth is ankylosed to the underlying bone, it is stabilized and therefore most acrodont species only have one generation of teeth (monophydont dentition)^10^. An exception might be the only extant Rhynchocephalia, tuatara (*Sphenodon punctatus*), which has some replacement ability and an acrodont dentition (Fig. 1)^9,11^, and some extinct reptiles such as *Opisthodontosaurus carrolli*^12^. In some lizards, both acrodont and pleurodont teeth are found within the same jaw. In the bearded dragon (*Pogona vitticeps,* Fig. 1), the anterior teeth are pleurodont and can replace, while the posterior teeth are acrodont and limited to one set^12,13^. From the fossil record, it appears that acrodont dentitions were derived from pleurodont. In extinct Chamaeleonidae and Agamidae (Fig. 1), a mix of pleurodont and acrodont attachment have been described^14^ similarly to extant agamas, again suggesting that pleurodonty is the basal state.

Given the distinct modes of attachment, it can be predicted that the development of the cervical loops might vary between the groups. We therefore aimed to determine the developmental processes contributing to the morphogenesis of acrodont dentition and to analyze possible symmetrical/asymmetrical features occurring on the labial and lingual sides of the cervical loop using embryos of the veiled chameleon (*Chamaeleo calyptratus*). 3D reconstructions of contrasted soft tissue in micro-CT enabled us to provide comprehensive morphometric analysis highlighting asymmetries in both tooth germ and bone development. Together with analysis of cell proliferation and microscopic and submicroscopic changes, we were able to investigate the early asymmetries and how they resolved into the symmetrical acrodont pattern.

## Material and methods

### Animals

Specimens of veiled chameleon (*Chamaeleo calyptratus)* and ocelot gecko *(Paroedura picta)* were obtained from a commercial breeder. Embryos and fetuses at different stages of development were collected to analyze the developmental progression. Eggs were incubated at the temperature of 27–29 °C. At selected time points, eggs were open, and embryos were euthanized by decapitation and fixed in 4% PFA at least overnight. To make our analysis easier for comparisons and interpretations, we focused only on the development of the lower jaw dentition and associated dentary bone morphogenesis.

All manipulations with chameleon and gecko embryos followed the specific rules for working with alive embryos as specified by the Central Commission for Animal Welfare of Ministry of Agriculture of the Czech Republic (§16a law No. 246/1992 Sb., for animal protection against cruelty). All analyses were performed in accordance with the guidelines, regulations and experimental protocols approved by the institutional and licencing committee including rules run by the Laboratory Animal Science Committee of the IAPG, v.v.i. (Liběchov, Czech Republic). Tissues were collected for analyses following embryo decapitation. No experiments were performed on embryos.

Embryo of tuatara (*Sphenodon punctatus*) was obtained from the Dendy collection at the King’s College London Museum of Life Sciences (specimen R, London UK) and an adult animal from the collection at the Department of Anatomy of Charles University (Prague, Czech Republic). Embryo of bearded dragon (*Pogona vitticeps*) originated from Prof. Tucker’s collection (London, UK). Material was used in accordance with museum guidelines and regulations. A phylogenetic tree was adapted from^15^ and^16^.

### Histology and immunohistochemical labelling

To prepare histological sections for immunohistochemical and histological analysis, specimens were fixed in 4% PFA. Tissues from older specimens were decalcified in 12.5% EDTA in 4% PFA at RT followed by paraffin embedding. Paraffin embedded tissues were cut to obtain serial transverse histological sections, which were stained with Haematoxylin–Eosin. Alternative slides were deparaffinised and rehydrated through a series of ethanol followed by antigen retrieval in a water bath (97 °C) in citrate buffer (pH = 6) for 20 min. Blocking serum was applied to the samples for 20 min to prevent unspecific binding of antibodies. Next, slides were incubated with primary antibodies PCNA (Cat. No. M0879, Agilent Dako, Santa Clara, California, USA) for 1 h or ST14 (Cat. No. ABIN277391, Antibodies Online, Aachen, German) overnight, respectively. The secondary antibodies (goat anti-mouse Alexa Fluor488, cat. No. A-11001, Thermo Fisher, USA) were applied for 30 min. DRAQ5 Fluorescent Probe Solution (cat. No. 62251, Thermo Fisher, USA) was used for the counterstaining. Images were taken on confocal microscope Leica SP8 using 20 × or 40 × (water immersion) objectives (Leica Microsystems, Germany) with Leica Application Suite software. Final photo processing was performed by software Imaris (Bitplane, Zürich, Switzerland).

For SOX2 detection, slides were incubated overnight with the primary antibody SOX2 (Cat. No. 2748, Cell Signaling, Leiden, Netherland). The secondary biotinylated anti-rabbit antibody (1:200, part of the ABC kit, Vectastain, Vector Laboratories, Burlingame, USA) and avidin–biotin complex (ABC kit, Vectastain, Vector Laboratories, Burlingame, USA) were applied for 30 min each followed by visualization with diaminobenzidine (DAB, Cat. No. K3468, DAKO, USA). Slides were counterstained by Haematoxylin to visualize nuclei. Tissues were analyzed and photographed using a Leica DMLB2 microscope (Leica Microsystems, Wetzlar, Germany).

PCNA-positive cells were counted at cap and early bell stages of chameleon odontogenesis. Samples from two different animals were used for each developmental stage. Seven tooth sections were used for cell counting at the cap stage, and sixteen at the early bell stage. Every tooth germ was divided into four distinct areas: the outer enamel epithelium of the lingual side of the cervical loop, the inner enamel epithelium of the lingual side of the cervical loop, the outer enamel epithelium of the labial and lingual sides of the cervical loop. Statistical significance in difference of PCNA-positive cell number between labial and lingual sides of the cervical loop were evaluated by paired t-test (Excel, Microsoft Corporation, USA).

### Transmission electron microscopy

Two samples from early developmental stages were fixed in 3% glutaraldehyde for 24 h. Mandibles were washed three times in 0.1 M cacodylate buffer and post-fixed in 1% OsO_4_ solution for 1 h. Samples were embedded in epoxy resin Durcupan. Semithin sections were stained with Toluidine Blue to evaluate general morphology. Ultra-thin sections were placed on formvar-coated nickel grids and contrasted with lead citrate and uranyl acetate. More details were published previously elsewhere^17^.

### Micro-CT measurement

Fourteen samples of embryonic chameleon jaws were selected for more detailed micro-CT analysis of hard tissue morphology (Fig. S1, Table S1). For the purpose of our study, we defined five developmental stages, with tooth development ranging from early mineralization to just prior to ankylosis, in order to highlight the dynamic relationship between the developing teeth and underlying bone.

In chameleons, differences in growth rate can occur, even between individuals from the same clutch of eggs. This is largely driven by differences in temperature, which can affect speed of development with lower temperature resulting in longer time to hatching^18,19^. In addition, the duration of embryonic diapause can differ between clusters of eggs, so that time of oviposition does not necessarily correlate with actual developmental stage^20^. Weight of embryos has been demonstrated to exhibit good correlation to developmental phase for staging of odontogenesis in mouse embryos^21^. We therefore used both number of embryonic days and embryonic weight to characterize our samples. Before scanning, all samples were embedded in 1% agarose gel in 1.5 ml Eppendorf tube in order to prevent the movement during the micro-CT scan. Samples were first scanned without staining in order to analyze the hard dental tissue and underlying jaw bone. For the evaluation of the soft tissue morphology and their relationship to hard tissues, five of the previously scanned samples were counterstained and underwent further analysis after re-scanning.

The staining protocol consisted of several dehydration steps including 30%, 50%, 70%, 80% and 90% ethanol solution—1.5 h in each concentration. After dehydration, samples were submerged in staining solution consisting of 1% iodine in 90% methanol for 16 h, after the staining step the samples were washed in 50% ethanol and embedded in 1% agarose.

The micro-CT scanning was performed using laboratory system GE phoenix v|tome|x L 240 (GE Sensing & Inspection Technologies GmbH, Germany), equipped with a 180 kV/15 W maximum power nanofocus X-ray tube and high contrast flat panel detector dynamic 41|100 (number of pixels: 4048 × 4048 px, pixel size 100 μm). The measurements were carried out in an air-conditioned cabinet (21 °C). The X-ray tube current of 200 µA, acceleration voltage of 60 kV and exposure time of 600 ms were common for all scanned samples. Number of images and voxel resolution were set individually for each sample depending on the size (Table S2). The tomographic reconstruction was done using the software GE phoenix datos|x 2.0 (GE Sensing & Inspection Technologies GmbH, Germany).

### Micro-CT data processing

Data from micro-CT scans of fixed unstained samples were analysed and measured in VGStudio MAX 3.3 licensed software (Volume Graphics GmbH, Germany, https://www.volumegraphics.com). Schematics of the measurements and plane establishment in the jaw are shown in Fig. S2 with individual teeth labelled in a rostro-caudal direction (Fig. S3—teeth shown for right side).

The following measurements and analyses were performed using the unstained sample datasets:

*Tooth to jaw distance* The distance between the teeth and the underlying bone was measured separately on the labial and lingual sides of the jaw in transversal sections through the jaw and tooth. These sections were defined by the polyline generated by placing points at the tips of teeth with the plane running perpendicular to the horizontal plane. For standardisation, the tooth to bone distance was measured at the highest point of each tooth.

*Tooth to jaw angle* The angle in which the tooth tilted towards the jaw (angle of inclination) was measured in the same plane as mentioned above.

*Wall thickness* Sphere based wall thickness analysis in VG studio software was used to assess the thickness of the examined samples by fitting spheres inside the sample in 3D space. The thickness was defined by the diameter of the fitted sphere.

Segmentation of the cervical loop in the iodine stained samples was carried out using Avizo 9.5 software. 3D models (in Stl format, https://www.fei.com/software/avizo3d/, Thermo Fisher Scientific, USA) of the cervical loop was then transferred in the VG studio software where the other analyses were performed.

The length of the cervical loop was measured on the labial and lingual side using transverse sections through the jaw (defined by the polyline) at the highest point of the tooth. The cervical loop extends around the forming dental papilla of the tooth germ as visualized in 3D by micro-CT (Fig. S4). However, in transversal sections through the head, we can only observe the lingual and labial sides of the loop and we use this nomenclature in the manuscript.

## Results

### Early asymmetrical growth of the labial and lingual part of the cervical loop in an acrodont dentition as revealed by microscopic and ultrastructural analysis

**Figure 2:**
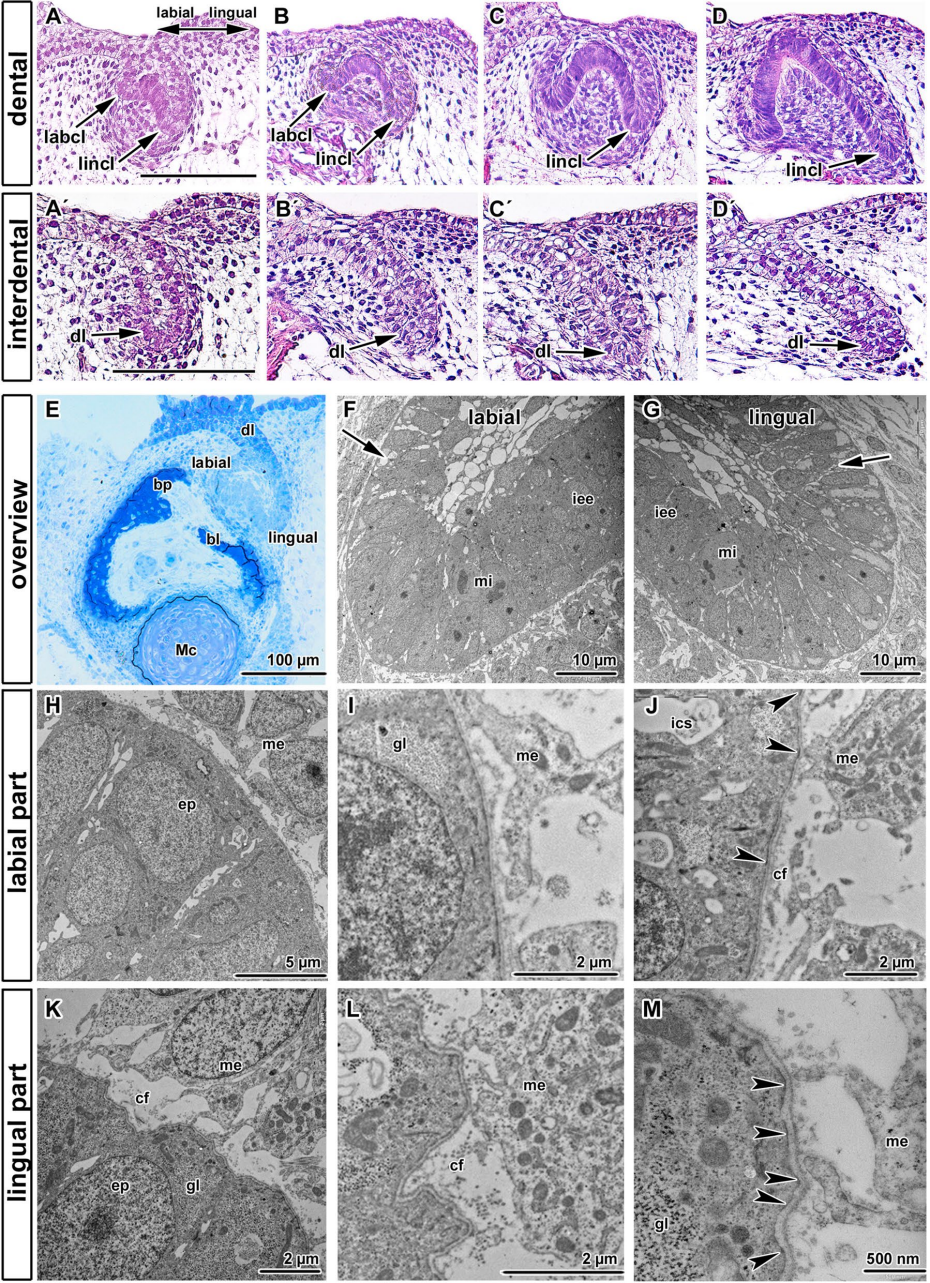
Labio-lingual asymmetry of enamel organ in early chameleon embryos. (**A**–**D**) Microscopic structure of developing tooth germ visualized by HE staining. (**A′**–**D′**) Interdental areas contain continuous dental lamina protruding in lingual direction. Late bud stage (**A**, **A′**), early cap stage (**B**, **B′**), late cap stage (**C**, **C′**) and early bell stage (**D**, **D′**). Notice asymmetrical growth of labial and lingual part of cervical loop starting from very early stages of tooth germ development. Scale bar = 50 µm. (**E**-**M**) Transmission electron microscopy of tooth germ at cap stage in chameleon revealed distinct differences between labial and lingual side of the cervical loop. (**E**) Overview of early cap stage on semithin section stained by Toluidine Blue. (**F**, **G**) Detailed overview of labial or lingual cervical loop with visible mitotic cells (mi). Intercellular spaces are larger in the outer enamel epithelium (arrows). (**H**, **I**) Details of the labial cervical loop with smooth basal membrane. Glycogen granules (gl) were situated in cells in upper part of the lingual and labial sides of the cervical loop (**K**). (**J**) Few hemidesmosomes were located in the labial part of the cervical loop (arrowheads). (**K**–**M**) In contrast, basal membrane of lingual cervical loop was distinctly folded with numerous collagen fibrils (cf) in its close proximity (**K**, **L**). Lamina lucida and lamina densa were strictly separated in this area and numerous hemidesmosomes were located in the lingual part of the cervical loop (**L**, **M**—arrowheads). *bl* bony lamella, *bp* bony pedicle, *cf* collagen fibrils, *dl* dental lamina, *ep* epithelial cell, *gl* glycogen, *ics* intercellular spaces, *iee* inner enamel epithelium, *labcl* labial part of the cervical loop, *lincl* lingual part of the cervical loop, *Mc* Meckel cartilage, *me* mesenchymal cell, *mi* mitosis.

From the early cap stage, the enamel organ of the chameleon tooth germ grew asymmetrically into the underlying mesenchyme with a longer extension of the cervical loop observed on the lingual side (Fig. 2A–D). Later, at the bell stage, asymmetry between the lingual and labial sides of the cervical loop was even more pronounced, with prominent elongation of the lingual part of the tooth germ into the mesenchyme (Fig. 2D).

In the interdental spaces, an epithelial outgrowth was visible along the entire jaw (Fig. 2A′–D′). The dental lamina extended in a lingual direction from the outer enamel epithelium of the tooth germ. In 3D, it was evident that the lingual part of the cervical loop was continuous with the interdental dental lamina and dental lamina, which extended along the jaw (Fig. S4), similar to the situation previously described in snakes^22^. During the course of development, the cervical loop expanded into the interdental areas and later, the individual teeth fused with the neighboring teeth (Fig. S4).

As chameleon teeth exhibited distinct asymmetrical development of the cervical loop, which is typically observed in pleurodont dentitions, we next investigated the ultrastructure of the lingual and labial sides of the loop (Fig. 2E–M). The inner enamel epithelium of both parts of the cervical loop was formed by several layers of small round nuclei (Fig. 2F,G). Minimal intercellular spaces were observed between the cells in the loops, in contrast to the cells in the outer enamel epithelium (Fig. 2F). Mitotic events were observed in both the labial and lingual sides of the cervical loop (Fig. 2F,G). Close interactions between cells on the epithelio-mesenchymal interface were identified at the edge of the cervical loop, with long cytoplasmatic processes of mesenchymal cells in direct contact with the basement membrane on both sides (Fig. 2H,K).

Despite these overall similarities, some distinct differences were observed, particularly at the epithelio-mesenchymal interface. The labial side of the cervical loop was surrounded by a smooth basal membrane without undulations (Fig. 2I,J). Separation of the basement membrane into the lamina lucida and lamina densa was distinct (Fig. 2I,J). In the area closely attached to the mitotically active cells, the basal membrane was fenestrated in several regions (data not shown). Large intercellular spaces with a size of up to 2 µm were distinct between the cells of the outer enamel epithelium on the labial side (Fig. 2J).

In contrast, the basal membrane of the lingual cervical loop cells was irregularly folded with distinct basal cells, which contained numerous hemidesmosomes on their membranes (Fig. 2J,M). Many primary lysosomes were located in the basal area of these epithelial cells. Mitochondria were small and scattered throughout the cellular cytoplasm and they represented the dominant organelle in these cells. Large amounts of glycogen were present in the basal areas of these cells (Fig. 2K,M). In contrast to the labial side, the lingual basement membrane was clearly subdivided into a lamina lucida and lamina densa (Fig. 2L,M). Numerous collagen fibrils were located directly beneath the basement membrane in the lingual part of the cervical loop (Fig. 2L), in contrast to the minimal numbers of fibrils in the labial part of the cervical loop (Fig. 2J). Long cellular processes of the mesenchymal cells protruded towards the epithelial cells (Fig. 2M). Numerous folds were found in the lingual part of the cervical loop and we observed significantly higher number of hemidesmosomes (Fig. 2J,M). The cervical loop of a tooth is therefore not a uniform structure with distinct differences on its labial and lingual sides.

### Asymmetrical extension is associated with differential proliferation between the labial and lingual sides of the cervical loop

Next, we analyzed the developmental processes that might be contributing to the asymmetrical extension of the cervical loop. We focused on cell proliferation at early stages of cervical loop elongation, at the cap and bell stage, before the dental lamina has separated from the outer enamel epithelium.

**Figure 3:**
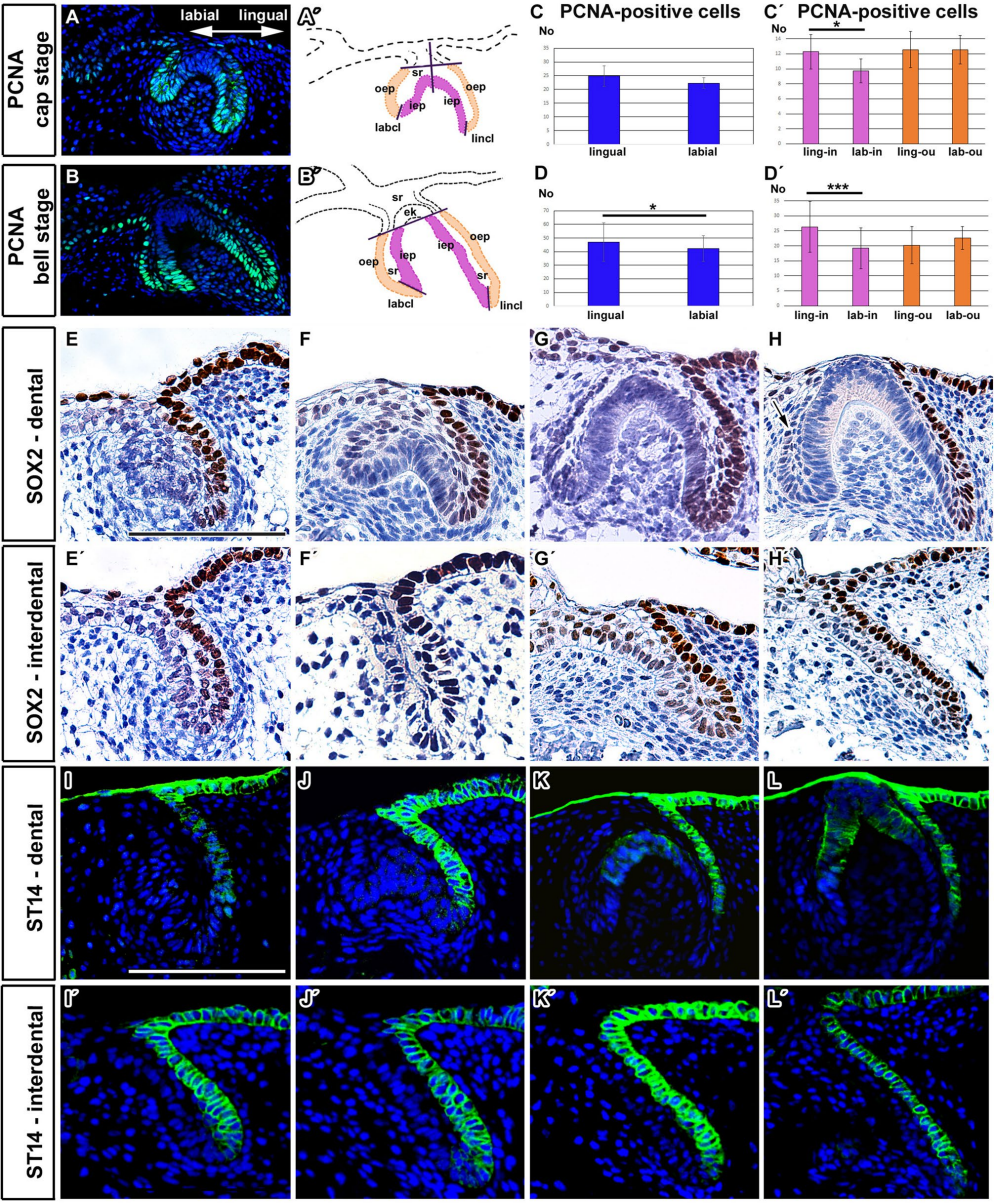
Differences in proliferation, SOX2 and ST14 expression in the labial and lingual side of the enamel organ in chameleon. (**A**–**D′**) Analysis of proliferation rate in chameleon tooth germs. (**A**, **B**) Localization of PCNA-positive cells in both labial and lingual sides of the cervical loop at the cap and bell stage. (**A′**, **B′**) Scheme with labeled areas where counting of PCNA-positive cells was performed. Outlined areas of proliferation analysis in the lingual and labial sides of the cervical loop. In order to detect differences in cell proliferation the number of PCNA-positive cells was counted separately for the inner (purple dotted line) and outer (orange dotted line) enamel epithelium of the labial and lingual cervical loop (separated by black line). (**C**, **D**) Graphs display numbers of PCNA-positive cells contributing to the lingual or labial part of the cervical loop. The lingual side of the cervical loop possesses more PCNA-positive cells in contrast to the labial side at the bell stage (**D**) but not at the cap stage (**C**). (**C′**, **D′**) Graphs display numbers of PCNA-positive cells contributing to the lingual (inner and outer enamel epithelium) and labial (inner and outer enamel epithelium) sides of the cervical loop. The lingual inner enamel epithelium contains more proliferating cells at the cap (**C**’) and bell (**D**’) stage. The graph values denote average ± s.d., ***p < 0.001, *p < 0.05 using a paired t-test. *ek* enamel knot, *iep* inner enamel epithelium, *labcl* labial side of the cervical loop, *lincl* lingual side of the cervical loop, *oep* outer enamel epithelium, *sr* stellate reticulum. (**E**–**H**) SOX2-positive cells in chameleon enamel organ with detailed view (**E′**–**H′**) on the lingual and labial part of the cervical loop and on the interdental area. (**E**, **F**) During early odontogenesis, strong SOX2 expression was detected predominantly in cells of the dental lamina situated on the lingual side of the developing tooth germ. (**G**, **H**) SOX2 expression persisted in the dental lamina cells even during early or late bell stages. SOX2 expression in the interdental area followed expression pattern in developing tooth germ with strong signal on its lingual side at early (**E′**, **F′**) or late developmental stages (**G′**, **H′**). Scale bar = 50 µm. (**I**–**L′**) ST14 expression during chameleon odontogenesis. (**I**, **J**) Localized lingual ST14 expression was detected predominantly at the late bud stage and early cap stage. (**K**, **L**) Later in development, ST14 positive cells were situated both in the enamel knot area and the lingual cervical loop. (**I′**–**L′**) ST14 expression was detected in the interdental area with same lingually located asymmetrical pattern at early (**I′**, **K′**) as well as late (**K′**, **L′**) developmental stages. Scale bar = 50 µm.

PCNA-positive cells were found to be located within the cervical loop at both stages analyzed (Fig. 3A,A′,B,B′). Significantly more PCNA-positive cells were observed on the lingual side of the loop at the bell stage, with no difference at the cap stage (Fig. 3C,D; Student pair t-test p = 0.172 at the cap stage and p = 0.037 at the bell stage). To evaluate the pattern of proliferation in more detail, the number of dividing cells in the inner and outer epithelial layers were counted for each side of the cervical loop (Fig. 3A′,B′). At both analyzed stages, there were significantly more PCNA-positive cells within the lingual inner enamel epithelium compared to the labial inner enamel epithelium (Student pair t-test p = 0.0488 at the cap stage and p = 0.0002 at the bell stage). In contrast, no difference was observed between the lingual and labial outer enamel epithelium at either stage (Student pair t-test p = 1.000 at the cap stage and p = 0.139 at the bell stage) (Fig. 3C′,D′).

### SOX2 is differentially expressed on the lingual and labial sides of the cervical loop similar to pleurodont dentitions

SOX2 expression is asymmetrically localized from early stages of mammalian and pleurodont reptile tooth development^8^, therefore we were interested if SOX2 expression would also be restricted to the lingual part of the cervical loop in the chameleon, where there is no tooth replacement and the successional dental lamina is only rudimentary.

In chameleon embryos, strong SOX2 expression was observed in the lingual part of the cervical loop through all analyzed stages (Fig. 3E–H; S5). The strongest signal was located in the lingual outer epithelium from very early stages of development (Fig. 3E–H). We detected SOX2 expression not only in the areas where a tooth would initiate but also in the interdental areas between teeth. Surprisingly, interdental dental lamina exhibited similar asymmetrical expression of SOX2, located on the lingual side of the epithelial protrusion (Fig. 3E′–H′) even though this region does not form teeth.

A few SOX2-positive cells were also found in the distal areas of the lingual inner enamel epithelium and lingual stellate reticulum (Fig. 3F,G,H). Even during early mineralization stages, SOX2-positive cells were found at the tip of the lingual part of the cervical loop overlapping with the inner enamel epithelium (Fig. S5A, A′). A few SOX2-positive cells were detected also in the stellate reticulum (Fig. S5). Later in development, SOX2 positive signal was located only in the outer layer of the dental lamina (Fig. S5).

The labial stellate reticulum and the inner enamel epithelium were SOX2-negative throughout development (Figs. 3G,H; S5), along with the differentiating odontoblasts and ameloblasts (Figs. 3G,H; S5).

### ST14 exhibits asymmetrical expression in the cervical loop with positive domain overlapping with SOX2-positive cells

To further determine labio-lingual differences, we analyzed the expression of ST14 (matriptase) during chameleon odontogenesis. Matriptase is a serine protease, which plays critical role in the maintenance of epithelial integrity in numerous tissues^23–25^, and is also expressed in the enamel knot during murine tooth development^17^. At early stages of development (bud to cap), ST14 was expressed on the lingual side of the cervical loop (Fig. 3I,J). Within interdental region, expression of ST14 also defined the lingual side of the lamina (Fig. 3I′–L′). At the bell stage, expression was maintained on the lingual side of the loop with a new domain of expression in the differentiating ameloblasts (Fig. 3K,L). Thus, the lingual side of the cervical loop is distinct molecularly from the labial side and shares an expression pattern with the connected interdental lamina.

### The cervical loop retains asymmetrical morphology during mineralization stages

**Figure 4:**
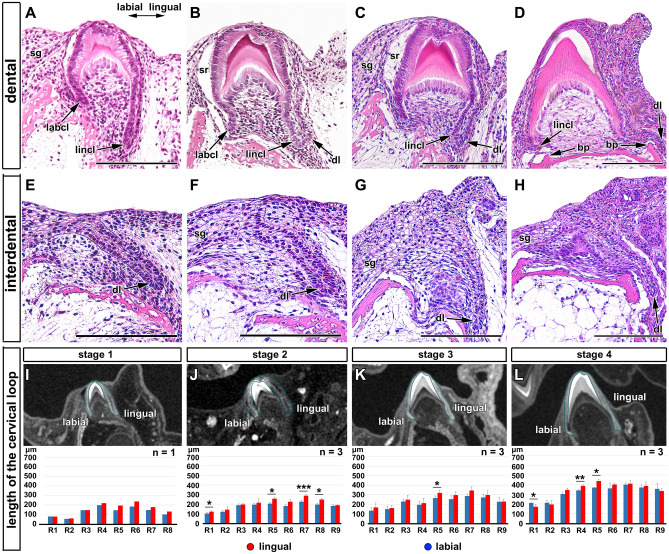
Labio-lingual asymmetry of enamel organ during mineralization stages in chameleon embryos. (**A**–**D**) Transversal histological sections through the central cusp of the tooth from early (**A**, **B**) to late (**C**, **D**) mineralization stage. Dental areas exhibiting distinct differences in the lingual and labial part of the cervical loop (arrows). (**E**–**H**) Detailed view on the interdental areas with dental lamina protruding lingually at early (**E**, **F**) and late (**G**, **H**) mineralization stages. *bp* bone pedicle, *dl* dental lamina, *labcl* labial cervical loop, *lincl* lingual cervical loop, *sg* salivary glands, *sr* stellate reticulum. Scale bars (**A**–**H**) = 50 µm. (**I**–**L**) Analysis of the length of the labial (blue) and lingual (red) parts of cervical loop revealed differences between the size of labial and lingual part of cervical loop at early (**I**, **J**) and late (**K**, **L**) mineralization stages. The lengths of labial and lingual part were not consistent along the jaw. The lingual part of the loop remained slightly longer throughout all analysed stages. R1 to R9 refer to the individual position of teeth on the right side of the jaw in order from the most rostral teeth (R1) to the tooth in the caudal area (R9). n = number of analysed mandibles for each stage. Pair t-test was used to analyze statistical significance of differences between labial and lingual part of the tooth. *p < 0.05, **p < 0.01, ***p < 0.001.

The labial part of the cervical loop remained shorter and directed lingually during stages of mineralization (Fig. 4A–D). The anlagen of the dental lamina appeared at the tip of the lingual part of the cervical loop as a protrusion of the outer enamel epithelium (Fig. 4C,D). Epithelial outgrowth in the interdental area was also directed lingually, similar to that observed at younger stages (Fig. 4E–H). This lingual direction fits with the differential proliferation pattern observed in the inner and outer epithelium of the cervical loop (Fig. 3C′,D′).

In the iodine contrasted micro-CT data, we were able to measure the length of the cervical loop in cross section through the center of the tooth (Fig. 4I–L). The analysis highlighted significant differences between the size of the labial and lingual part of cervical loop even in these later stages (Fig. 4I–L). The differences in length of the lingual and labial part of the cervical loop were not consistent along the jaw, with the anterior teeth being more symmetrical in comparison to the teeth that developed in the middle of the lower jaw, where the most significant differences in length were observed (Fig. 4I–L).

### Asymmetric association of the tooth and forming bone

**Figure 5:**
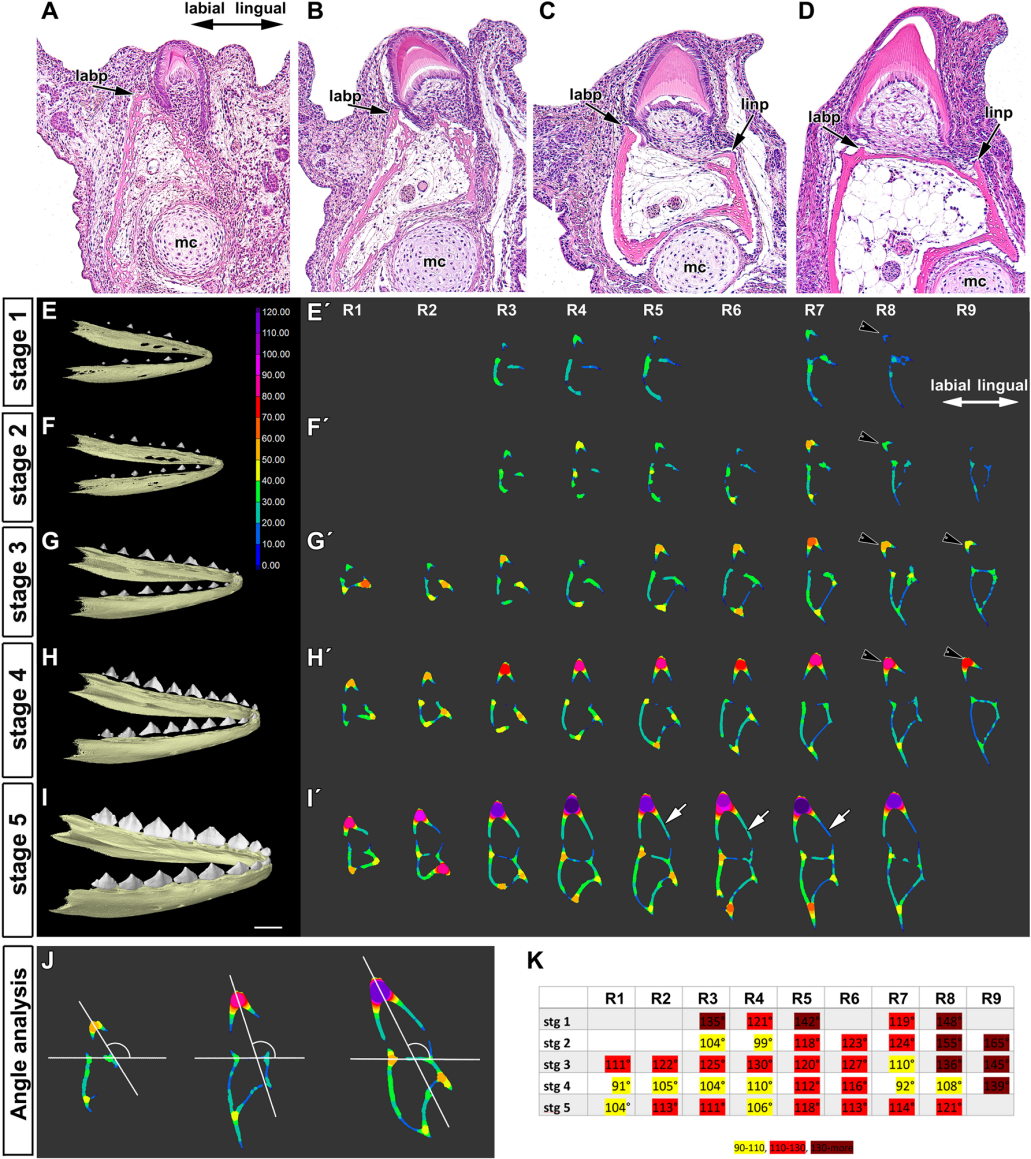
Tooth-bone relationship during prehatching development of chameleon. (**A**–**D**) Position of tooth and underlying bone during tooth germ development visualized by Haematoxylin–Eosin staining. (**A**) At early mineralization stage, labial bony pedicle (*labp*) was formed in close proximity to the labial part of the cervical loop. (**B**) Later in development, lingual bony pedicle (*linp*) approached extending lingual part of the cervical loop. (**C**) Once the bony pedicles were fully developed, bony lamellae were starting to form. (**D**) Spatial relationship between tooth and bone just before the attachment, when cells of the cervical loop were in direct contact with bone forming cells. *mc* Meckel cartilage. Scale bar = 50 um. (**E**–**I**) Lateral view on the 3D images of whole lower jaws and teeth in chameleon embryos (**E′**–**I′**). Transversal sections through the central area on each tooth with applied wall thickness analysis. Analysis was performed at five different stages: stage 1 (**E**, **E′**), stage 2 (**F**, **F′**), stage 3 (**G**, **G′**), stage 4 (**H**, **H′**) and stage 5 (**I**, **I′**). Micro-CT imaging revealed that the bone lamellae were thicker on the labial part of the jaw in all analysed developmental stages of chameleon embryo and in the areas of future lamellae branching/fusion. The initiation of bony pedicles mineralization was initiated on the labial side of the dentary bone and later during development progressed to the lingual side. R1 to R9 refer to the individual position of teeth on the right side of the jaw in order from the most rostral teeth (R1) to the tooth in the caudal area (R9). The unit in the colour scale is in µm. White arrows indicate visibly longer hard tissue of the lingual cervical loop. (**J**, **K**) Analysis of the tooth tilt towards the jaw in the transversal section of the teeth. (**J**) Measurements performed for selected teeth: tooth R7 at stage 2, tooth R8 at stage 4, tooth R5 at stage 5. (**K**) Analysis of the angle between dorsal bony lamellae and tooth axis revealed that the early stages of teeth were located with larger angle to the dorsal bony lamellae (**K**), labelled by black arrows in **E′**–**H′**. Moreover, caudal teeth were inclined in the jaw under different angle in comparison to the rostral teeth. *R* right tooth on the lower jaw.

While adult chameleon teeth are located above the bone, during late development the mineralized tooth germs were positioned at an angle to the forming jawbone (Fig. 5A–D). Moreover, the shape of the bone and the cervical loop did not correspond, indicating that the cervical loop did not grow directly towards the bone (Fig. 5A–C). As the teeth formed, the bony pedicles were initiated and extended upwards (Fig. 5D).

Based on histological analyses, development of the dentary bone, which holds the teeth, also appeared to develop asymmetrically along the labio-lingual axis. To assess this, we analyzed dentary bone morphology in more detail using 3D reconstruction. Micro-CT imaging revealed that mineralization of the dentary bone was enhanced on the labial side of the lower jaw (Fig. 5E–I, E′–I′). As the tooth-bone junction started to develop, bony lamellae mineralized just above Meckel’s cartilage, and these lamellae, together with the bony spicules located more lingually, were then remodeled to correspond to the shape of the cervical loop (Fig. 5H′,I′). Distinct differences in bone thickness were evident along the lower jaw (Fig. 5E′–I′). The bone appeared thicker on the labial side of the jaw at all developmental stages and also in the areas where the lamellae would later fuse (Fig. 5E′–I′).

Micro-CT of hard tissues was used to determine the relative position of each tooth in relation to the underlying bones during development (Fig. S3; movable models of individual stage Fig. S6–S10). The spatial relationship of tooth and bone was not synchronized during these mineralization stages (Fig. 5E′–I′). Measurement of the angle between the dorsal bony lamellae and tooth axis (Fig. 5J) revealed that all teeth were inclined labially on the jaw. Caudal teeth were positioned at a greater angle to the dorsal bony lamellae and exhibited a larger displacement (Fig. 5K).

### Tooth-bone distances were asymmetrical with larger distances on the lingual side

**Figure 6:**
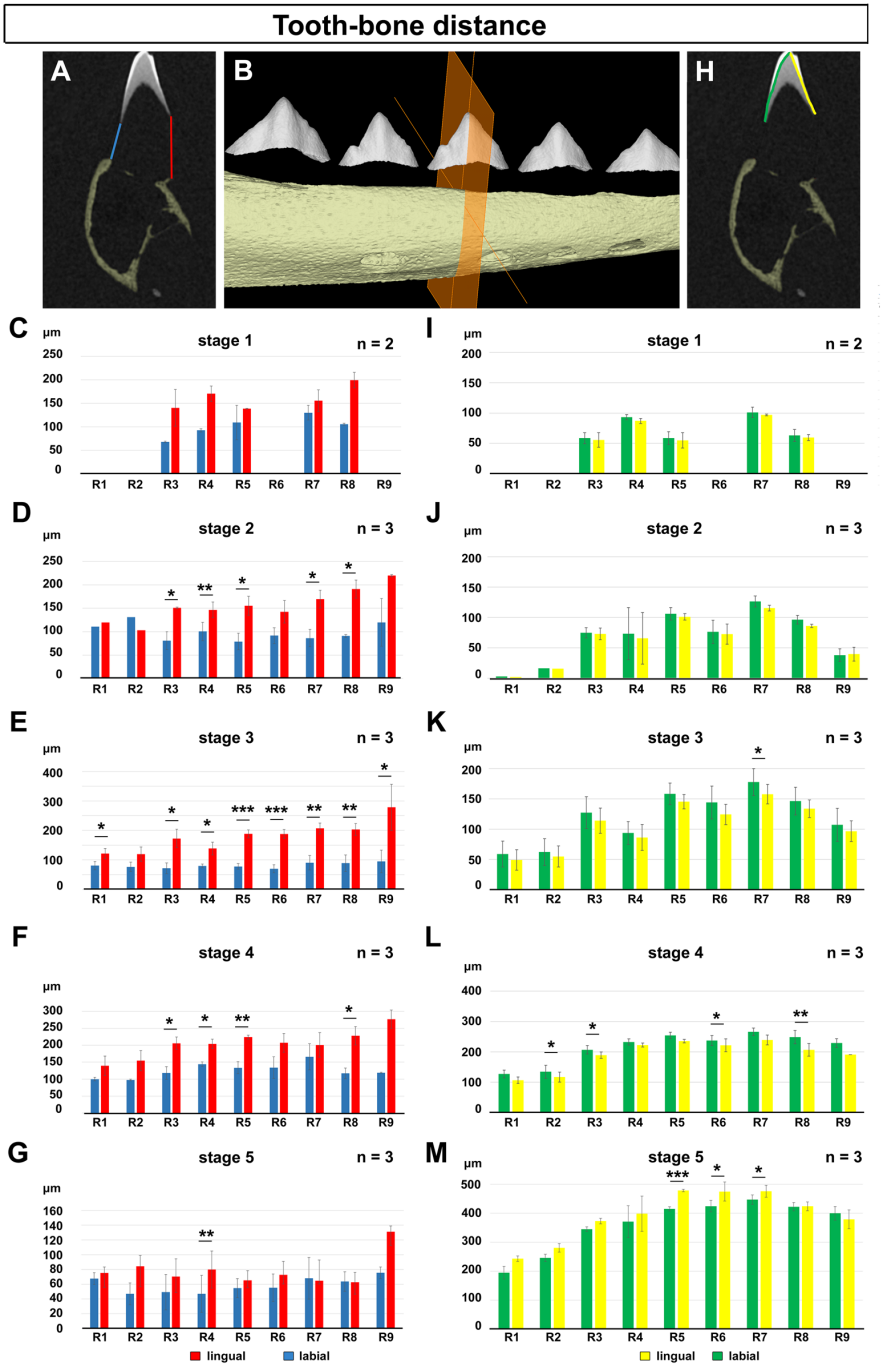
Tooth-bone distance and dentin deposition in chameleon. (**A**–**G**) Tooth to bone distance was measured on the transversal section through the central cusp (**B**). The distance was measured separately on the labial (blue) and lingual (red) side between the dentin and the nearest edge of the bony lamellae (**A**). Tooth-bone distance was found to be larger in the lingual side of the cervical loop at all analysed stages. (**H**–**M**) Analysis of dentin deposition on the labial (green) and lingual (yellow) side of the tooth germ. Analysis was performed at five different stages: stage1 (**C**, **I**), stage 2 (**D**, **J**), stage 3 (**E**, **K**), stage 4 (**F**, **L**) and stage 5 (**G**, **M**). n = number of analysed mandibles for each stage. Pair t-test was used to evaluate statistical significance of differences between labial and lingual part of the tooth. *p < 0.05, **p < 0.01, ***p < 0.001.

As the hard tissues of the tooth and bone need to come together to form an attachment, we investigated the distance between the dentin and the bone on both sides of the tooth at several developmental stages (Fig. 6A, B). Despite the fact that the lingual loop was considerably longer than the labial loop, the distance between the bone and dental hard tissue was larger on the lingual side in comparison to the labial side (Fig. 6C–G). For those stages where we had enough specimens, the differences were confirmed as statistically significant, although there was variation between tooth positions. By stage 5, the differences were generally not significant, suggesting the two sides of the bone and tooth had aligned, however, we identified much larger variation at this stage, which may have influenced the analysis (Fig. 6G). Overall, the largest gap on the lingual side was evident at stage 3 (up to 400 µm), reducing to about 80 µm by stage 5 (Fig. 6E–G).

### Dental hard tissues were deposited symmetrically, independent of length of the cervical loop

As we observed asymmetrical morphology of the soft dental tissues and bone deposition, including obvious differences in tooth-bone distances in the lingual and labial areas, we asked if deposition of hard dental tissue followed this pattern. Therefore, we analyzed the differences in the length of the hard tissue deposited on the lingual and labial sides of the cervical loop (Fig. 6H). We expected that odontoblast and ameloblast differentiation might follow the growth of the cervical loop. Bone deposition and soft dental tissues exhibited asymmetrical morphology from the beginning of development, the first deposition of dentin was symmetrical with no obvious differences between the labial and lingual sides (Fig. 6I,J). This symmetry was largely maintained at stage 3, with only one tooth in the jaw showing a statistical difference (Fig. 6K). At stage 4 and 5, this symmetry was briefly disrupted, with more dentin production initially on the labial side of the tooth (Fig. 6L) followed by higher production on the lingual side by stage 5 (Fig. 6M). Our findings therefore reveal the absence of an association between the length of the cervical loop and the progression of cell differentiation.

### Cervical loop development varies across reptiles with acrodont dentitions

**Figure 7:**
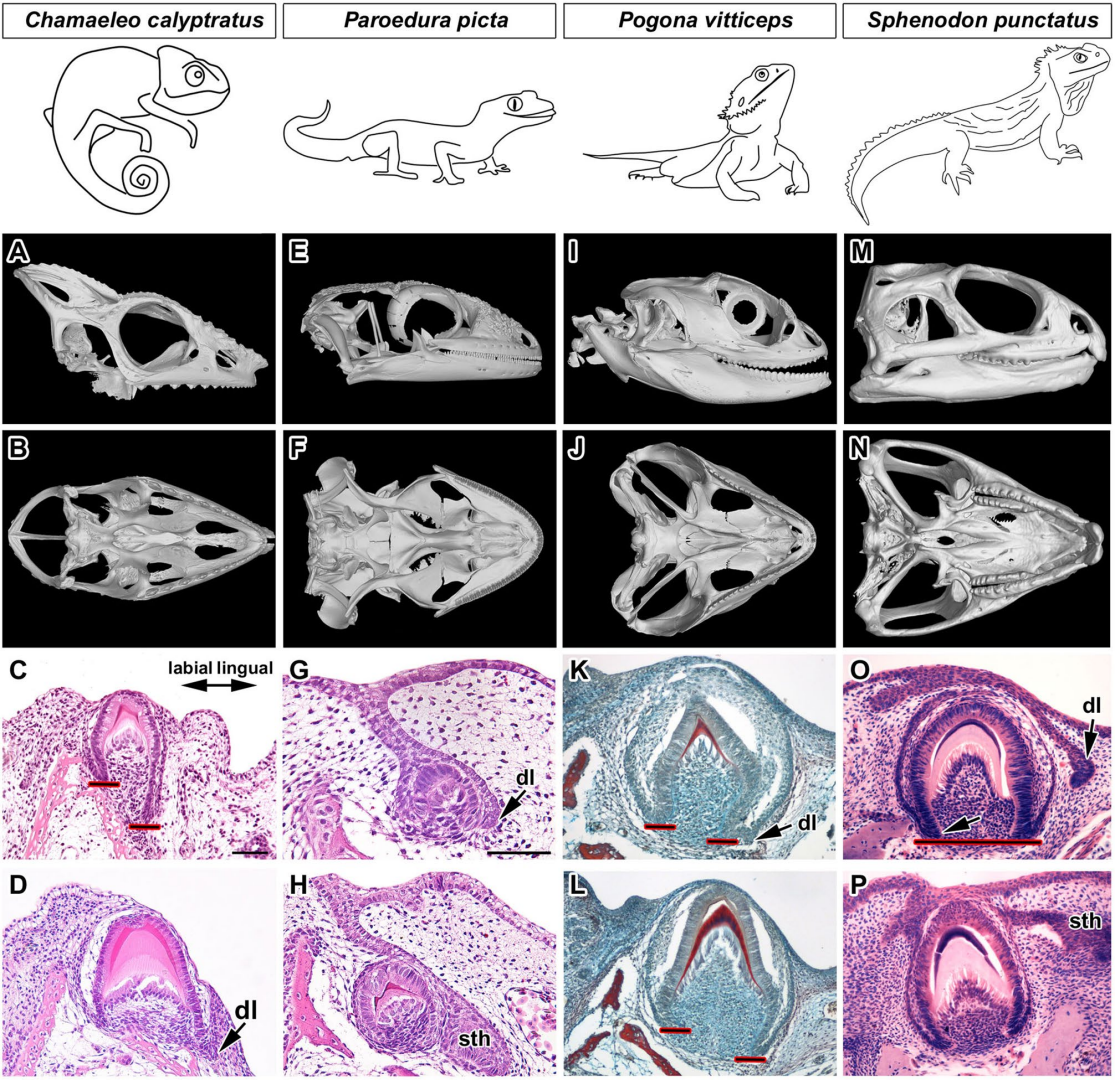
Comparison of tooth symmetry/asymmetry of selected reptilian species. (**A**–**D**) *Chamaeleo calyptratus*. (**A**, **B**) Lateral and palatal view of the skull of *Chamaeleo calyptratus* displaying complex teeth located in one row on the upper jaw. (**C**, **D**) Asymmetrical tooth germs at two different developmental stages. (**E**–**H**) *Paroedura picta*. (**E**–**F**) Lateral and palatal view of the skull of *Paroedura picta* displaying complex teeth located in one row on the upper jaw. (**G**, **H**) Asymmetrical tooth germs at two different developmental stages. (**I**–**L**) *Pogona vitticeps*. (**I**, **J**) Lateral and palatal view of the skull of *Pogona vitticeps* displaying complex teeth located in one row on the upper jaw. (**K**, **L**) Asymmetry in the development of the cervical loop was evident in acrodont tooth germs developing in the posterior area (**L**) of the jaw during mineralization stages at day 55 post-oviposition. (**M**–**P**) *Sphenodon punctatus*. (**M**, **N**) Lateral and palatal view of the skull of *Sphenodon punctatus* displaying complex teeth located in two rows posteriorly on the upper jaw. The teeth of the lower jaw fit into the groove between the two rows of teeth on the upper jaw. (**O**, **P**) Symmetrical tooth germs were observed in both the posterior (**O**) and more anterior (**P**) regions of the jaw. Specimen R, 5 months old. *dl* dental lamina, *sth* successional tooth, *black line with red outlines* ending of the cervical loop. Scale bar = 50 µm.

The described asymmetrical growth of the cervical loop in chameleon (Fig. 7A–D) appears to represent a common condition, which has been described in many other reptilian species with pleurodont attachment^1,5,26^. In the pleurodont gecko (Fig. 7E,F), the embryonic lingual part of the cervical loop was longer and thinner during all prehatching stages and the dental lamina cleaved off from the lingual outer enamel epithelium at the bell stage (Fig. 7G,H), similar to that previously described^5,13,27^.

The fact that both an acrodont (chameleon) and pleurodont (gecko, python) reptiles have asymmetrical cervical loops might suggest this state as a general feature of reptile dentitions. To investigate this further we assessed the development of the cervical loop in two other acrodont species, the bearded dragon (*Pogona vitticeps*) and tuatara (*Sphenodon punctatus*). In the caudal acrodont teeth of the bearded dragon (Fig. 7I,J), we observed a shorter cervical loop on the labial side during mineralization (Fig. 7K,L), which was evident in different regions of the jaw^13,28^. Agreeing with previous reports, a small rudimentary dental lamina was observed budding off from the lingual side of the cervical loop in the acrodont teeth (Fig. 7K). The bearded dragon, like the chameleon, therefore, has retained asymmetrical cervical loops, despite having an acrodont dentition in the majority of the jaw. In contrast, in the tuatara (Fig. 7M,N), which unlike the other reptiles described is a Rhyncocephalia, the two sides of the cervical loop in section appeared very symmetrical, evident in both anterior as well as in posterior teeth (Fig. 7O,P). Interestingly, the relationship of the dental lamina and primary tooth appeared different in tuatara, with the successional lamina extending from an area close to the oral surface rather than being closely associated with the lingual side of the cervical loop (Fig. 7O).

## Discussion

### Labio-lingual asymmetries in cervical loop morphology

Shaping of the tooth-bone interface, and the spatial relationship between these two tissues, has not previously been well described in non-mammalian species. Here we have focused on the processes contributing to the formation of the symmetrical morphology of dental tissues in the chameleon. Our analysis has revealed that the acrodont teeth of chameleons initially form asymmetrically, with differential extension of the cervical loop on the lingual and labial sides. This asymmetry was evident from early stages of tooth development but later resolved to create the strict labio-lingual symmetry observed in adult teeth.

The asymmetry observed in the developing chameleon and bearded dragon, mirrors that observed in the gecko and snake, which have a pleurodont attachment. Our findings, therefore, support the view that pleurodonty is a plesiomorphic character and has been adapted to create an acrodont attachment. The differences between labial and lingual sides of the cervical loop seems to be both morphologically and functionally tightly associated with development of the dental lamina, even in chameleon where a replacement dentition is not formed and only a rudimental successional dental lamina is visible. In contrast, in the tuatara, where the dental lamina was less tightly associated with the lingual side of the cervical loop, the loop was symmetrical. The asymmetrical nature of developing teeth in squamates is, therefore, likely to be a consequence of the close relationship of the epithelium on the lingual side of the cervical loop and the dental lamina. In contrast, we observed no clear link between symmetrical/asymmetrical growth of the cervical loop and the type of attachment or whether a replacement tooth formed or not.

Variations in size of the labial and lingual parts of the cervical loop have also been observed in mammalian species. Very small asymmetries are visible at early stage of mice molar development, where the lingual side of the cervical loop is slightly longer and thinner than the labial side^29^. More obvious disparities have been widely studied in rodent incisors, where the lingual side of the cervical loop is less robust in comparison to the labial cervical loop, which houses a stem cell population for regeneration^30^. This asymmetry is already visible from the bud stage of tooth development and has a clear functional consequence, creating the asymmetry in enamel formation in the tooth. In contrast, it appears to be no functional difference in the two sides of the cervical loop in the chameleon.

The asymmetrical morphology was confirmed at the ultrastructural level, with differences in cell junctions and basal membrane morphology. The basal membrane of the lingual cervical loop cells contained numerous hemidesmosomes. Moreover, numerous folds were located in the lingual side of the cervical loop while the labial side was smooth. Comparable folding of basal membrane was also observed in mouse molars during the bending of the inner enamel epithelium at the bell stage^29^. Desmosomes and hemidesmosomes can provide mechanical integrity to the dental epithelial tissues^31^ and differences in their distribution might be involved in remodeling of the cervical loop. Abundant cells packed with glycogen were observed in the outer enamel epithelium with higher amounts of glycogen granules on the lingual side of the cervical loop. It was previously suggested that highly glycogen packed cells undergo cell degeneration^29^. However, in the case of the chameleon, these cells seemed to be very metabolically active and did not exhibit other features of cellular regression. Increased glycogen storage has also been observed during transition from preameloblasts to ameloblasts^32,33^. Moreover, some glucose transporters (GLUT1) with a high affinity for glucose exhibit strong expression in active and proliferating cells in mice^33^. Therefore, in chameleon, the distribution of large glycogen deposits in cells of the early tooth germ may be associated with the high proliferation status of the epithelial cells in the cervical loop area.

### Labio-lingual difference in gene expression

Previously, it was reported that even small alteration in cellular dynamics can finely tune tooth size^34^, therefore we decided to analyze possible differences in the number of proliferating cells in the labial and lingual sides of the cervical loop. A significant difference was observed between the lingual and labial sides of the loop at the bell stage, with more proliferating cells on the lingual side. In contrast, no difference was observed in the number of proliferating cells within the loop at the cap stage. However, when the epithelium was divided into inner and outer enamel epithelium, significantly more proliferating cells were observed within the lingual inner enamel epithelium compared to the labial inner enamel epithelium at both the cap and bell stages. The asymmetry in the loops is therefore likely to be driven, in part, by the difference in proliferation of the inner enamel epithelium on either side of the loop. This arrangement would also be predicted to result in an overall extension of the cervical loop in a lingual direction, agreeing with the histology and soft tissue micro-CT data. Although significant differences in proliferation were highlighted, particularly at the bell stage, other mechanisms are likely to also drive the differential extension of the cervical loop. At both the cap and bell stages obvious shape differences were identified between the two sides of the cervical loop, where the labial side was short and thick while the lingual side was thinner and elongated. Processes such as cell rearrangement and intercalation may therefore help to drive the changes in cervical loop shape.

Localized SOX2 expression as a stem cell marker has been previously described in the lingual side of the tooth germ, in the dental lamina of reptiles as well as in the dental lamina during serial addition of mammalian molars^8^. SRY-related HMG box-containing transcription factor-2 (SOX2) is a key factor that plays an important role in maintaining pluripotency of stem cells^35^. Expression of SOX2 has been previously found to be restricted in the stem cell niche and SOX2 is involved in successional dental lamina development in a number of different vertebrate species^8^. Similarly, we observed SOX2 expression largely restricted to the lingual side of the tooth germ, despite the fact that there is no replacement and the dental lamina is only rudimentary^36^. The early presence of SOX2, therefore, does not indicate whether a replacement tooth will form or not. SOX2 has previously been found to control and promote proliferation, with the inhibition of SOX2 leading to suppression of cell proliferation and invasion^37^. However, we identified no difference in proliferation between the SOX2-positive outer lingual loop and the SOX2-negative outer labial loop, suggesting SOX2 does not boost proliferation at the cap and bell stages.

Expression of SOX2 was continuous along the jaw in the chameleon, similar to that recently described in the minipig^38^. In animals with a dental lamina running along the jaw the expression of SOX2 may therefore act to promote formation of an interdental lamina. Previously, it has been proposed that *Sox2* expression relates to the competence of the interdental epithelium for tooth initiation^39^.

Previously, we revealed that matriptase (also known as ST14, TADG15, or epithin) is expressed in the enamel knot at the cap stage but also exhibits distinct expression in the tooth germ of chameleons^17^. Here, we asked if the expression in the tooth germ is restricted to specific part of the dental loop and how the expression pattern appears in the interdental areas. We found ST14 signal located only on the lingual side of the cervical loop. In the interdental areas, ST14 expression was also restricted to the lingual side. The role of ST14 in odontogenesis has not been studied yet but its expression makes it an interesting gene for further study. As matriptase displays trypsin-like serine protease activity and it is able to cleave and activate numerous substrates, it is possible that matriptase may regulate local signaling that contributes to the formation and function of the dental epithelium.

### Dental hard tissue deposition does not follow the pattern of the cervical loop

Adult chameleon teeth are typically located just above the dentary bone and ankylosed to the tip of bony pedicles^26,40^. We expected that odontoblast and ameloblast differentiation might follow the growth of the cervical loop, however, despite the asymmetrical extension of the loop, the deposition of dental hard tissue was symmetrical. This was in contrast to the maintained asymmetry typical for pleurodont dentition in the gecko, which is achieved by extended dentin deposition on the lingual side during odontogenesis^5^. Loop extension and the extent of hard tissue deposition is therefore independently regulated in the chameleon. How cells recognize the necessity to continue to differentiate or prevent their progression along the cervical loop would be an interesting area to analyse in future studies. The generation of a symmetrical structure from an asymmetrical template, as observed in the chameleon tooth, may additionally shed light on the creation of other symmetrical structures in the body^41^.

## Conclusion

Overall, our studies highlight the close relationship between the lingual side of the cervical loop and the dental lamina in squamates, both morphologically and molecularly. We show that it is not necessary to have a symmetrical cervical loop to form a symmetrical tooth, but that this can be generated by asymmetrical development in the tooth and bone, and finally we suggest that symmetrical teeth in squamates evolved from asymmetrical pleurodont teeth.

## Supplementary Information

Supplementary Tables.

Supplementary Figure S1.

Supplementary Figure S2.

Supplementary Figure S3.

Supplementary Figure S4.

Supplementary Figure S5

Supplementary Figure S6

Supplementary Figure S7

Supplementary Figure S8.

Supplementary Figure S9.
