# Supplementary material for: Coordinated labio-lingual asymmetries in dental and bone development create a symmetrical acrodont dentition
Source: Sci Rep. 2020 Dec 16;10:22040. doi: 10.1038/s41598-020-78939-2 (PMC7745041; doi:10.1038/s41598-020-78939-2)
Supplement: Supplementary file 1 — Supplementary Tables. [file 41598_2020_78939_MOESM1_ESM.doc]

**Co-ordinated labio-lingual asymmetries in dental and bone development create a symmetrical acrodont dentition**

Kavková M.^1^*, Šulcová M.^2^*, Dumková J.^3^, Zahradníček, O.^4^, Kaiser J.^1^, Tucker A.S.^5^, Zikmund T.^1^, Buchtová M.^6^
 
^1^*Central European Institute of Technology, Brno University of Technology, Brno, Czech Republic*
*^2^Department of Experimental Biology, Faculty of Science, Masaryk University, Brno, Czech Republic*

*^3^Department of Histology and Embryology, Faculty of Medicine, Masaryk University, Brno, Czech Republic*

*^4^ Department of Developmental Biology, Institute of Experimental Medicine, v.v.i., Czech Academy of Sciences, Prague, Czech Republic*

*^5^ Centre for Craniofacial and Regenerative Biology, Floor 27 Guy's Tower, Guy's Hospital, King's College London, London Bridge, London, UK*

*^6^ Laboratory of Molecular Morphogenesis, Institute of Animal Physiology and Genetics, v.v.i., Czech Academy of Sciences, Brno, Czech Republic*

* Authors contributed equally

**SUPPLEMENTARY FIGURES**

**Figure S1: Micro-CT view of used embryos for morphometric analyses**

Dorso-lateral view on the lower jaw of individual animals used for detailed morphometric analyses of all five stages. The rostral tip of the jaw is located on the right side.

**Figure S2: Micro-CT data processing in individual steps**

(A) The polyline (green line) was defined by placing points at the tip of all teeth in the lower jaw. By using the polyline, the new plane of sections was constructed (magenta). The plane of transversal section through the tooth (orange) was designed perpendicularly to the magenta plane. (B) Transversal section through the tip of the tooth (orange plane). (C) Longitudinal section through the teeth and jaw in magenta plane.

**Figure S3: Micro-CT of the chameleon lower jaws with individual teeth labelled**

Lateral view on the right side of the lower jaw for individual stages. Teeth are labeled from the most rostral tooth (R1) up to the most caudal tooth (R9).

**Figure S4: 3D image of segmented soft dental tissues from micro-CT data**

Soft tissue of the cervical loop was segmented in iodine stained samples of the lower jaws and 3D reconstruction is shown from the ventral aspect (dental papilla is open). The rostral part of the dental lamina is located on the right side. Lingual (lincl) and labial (labcl) sides of the cervical loop are labeled by white arrows. Interdental area of the dental lamina connecting individual teeth is labeled by a red arrowhead. Only the right sides of the dental lamina are segmented here. Wall thickness analyses display differences in the thickness of the cervical loop with the thinnest area (blue) located in the labial part of the cervical loop.

**Figure S5:** **SOX2 expression at mineralization stages in chameleon**

(A-D) Distribution of SOX2-positive cells during development of the chameleon enamel organ. (A´-D´) Detailed view on the lingual and labial side of the cervical loop of older mineralization stages. During later stages of odontogenesis, strong SOX2 expression was detected predominantly in cells of the dental lamina situated on the lingual side of the developing tooth germ. Scale bar = 50 µm.

**Figure S6: 3D view of chameleon bone in micro-CT at stage 1** (movable PDF)

Three different views on the bone or teeth at stage 1 can by changed by clicking first on bottom pictures and then by rotating the structure in 3D in upper picture. New version of Adobe Acrobat Reader is necessary and 3D imaging needs to be allowed.

**Figure S7:** **3D view of chameleon bone in micro-CT at stage 2** (movable PDF)

Three different views on the bone or teeth at stage 2 can by changed by clicking first on bottom pictures and then by rotating the structure in 3D in upper picture. New version of Adobe Acrobat Reader is necessary and 3D imaging needs to be allowed.

**Figure S8:** **3D view of chameleon bone in micro-CT at stage 3** (movable PDF)

Three different views on the bone or teeth at stage 3 can by changed by clicking first on bottom pictures and then by rotating the structure in 3D in upper picture. New version of Adobe Acrobat Reader is necessary and 3D imaging needs to be allowed.

**Figure S9:** **3D view of chameleon bone in micro-CT at stage 4** (movable PDF)

Three different views on the bone or teeth at stage 5 can by changed by clicking first on bottom pictures and then by rotating the structure in 3D in upper picture. New version of Adobe Acrobat Reader is necessary and 3D imaging needs to be allowed.

**Figure S10:** **3D view of chameleon bone in micro-CT at stage 5** (movable PDF)

Three different views on the bone or teeth at stage 5 can by changed by clicking first on bottom pictures and then by rotating the structure in 3D in upper picture. New version of Adobe Acrobat Reader is necessary and 3D imaging needs to be allowed.

**SUPPLEMENTARY TABLES**

**Table S1:** List of analyzed samples for micro-CT

| **Sample** | **Date of clutch laying** | **Date of embryo collection** | **Age of embryo (days)** | **Weight of the egg (g)** | **Weight of the embryo (g)** |
| --- | --- | --- | --- | --- | --- |
| Stage 1 (CH379) | 12.01.2019 | 20.5.2019 | 128 | 1.23 | 0.25 |
| Stage 1 (CH312) | 8.5.2018 | 31.8.2018 | 115 | 1.18 | 0.29 |
| Stage 2 (CHM313) | 08.05.2018 | 31.08.2018 | 115 | 1.31 | 0.37 |
| Stage 2  (CHM7) | 19.2.2020 | 30.6.2020 | 132 | 2.69 | 0.35 |
| Stage 2  (CHM15) | 19.2.2020 | 8.7.2020 | 140 | 3.2 | 0.4 |
| Stage 3  (CHM389) | 12.01.2019 | 27.5.2019 | 135 | 1.77 | 0.4 |
| Stage 3  (CHM13) | 19.2.2020 | 8.7.2020 | 140 | 3.15 | 0.48 |
| Stage 3  (CHM14) | 19.2.2020 | 8.7.2020 | 140 | 3.06 | 0.4 |
| Stage 4 (CHM397) | 12.01.2019 | 6.6.2019 | 145 | 1.47 | 0.44 |
| Stage 4 (CHM405) | 12.1.2019 | 11.6.2019 | 150 | 1.81 | 0.45 |
| Stage 4 (CHM326) | 21.6.2018 | 14.11.2018 | 146 | 1.62 | 0.55 |
| Stage 5 (CHM327) | 21.06.2018 | 14.11.2018 | 146 | 1.81 | 0.65 |
| Stage 5 (CHM411) | 12.1.2019 | 19.6.2019 | 158 | 2.1 | 0.48 |
| Stage 5 (CHM25) | 19.2.2020 | 4.8.2020 | 167 | 3.25 | 0.86 |

**Table S2:** Parameters of micro-CT scans for individual stages

| **Sample** | **Number of images** | **Scanning time [min]** | **Voxel resolution [µm]** |
| --- | --- | --- | --- |
|
| Stage 1 | 2500 | 105 | 2.5 |
| Stage 1 - stained | 2500 | 105 | 2.5 |
| Stage 2 | 2400 | 105 | 3.5 |
| Stage 2 - stained | 2400 | 105 | 3.5 |
| Stage 3 | 2400 | 105 | 3 |
| Stage 3 - stained | 2400 | 105 | 3 |
| Stage 4 | 2400 | 105 | 3 |
| Stage 4 - stained | 2400 | 105 | 3 |
| Stage 5 | 2200 | 85 | 3 |
| Stage 5 - stained | 2200 | 85 | 3 |
