# Supplementary figures and images for: Coordinated labio-lingual asymmetries in dental and bone development create a symmetrical acrodont dentition
Source: Sci Rep. 2020 Dec 16;10:22040. doi: 10.1038/s41598-020-78939-2 (PMC7745041; doi:10.1038/s41598-020-78939-2)

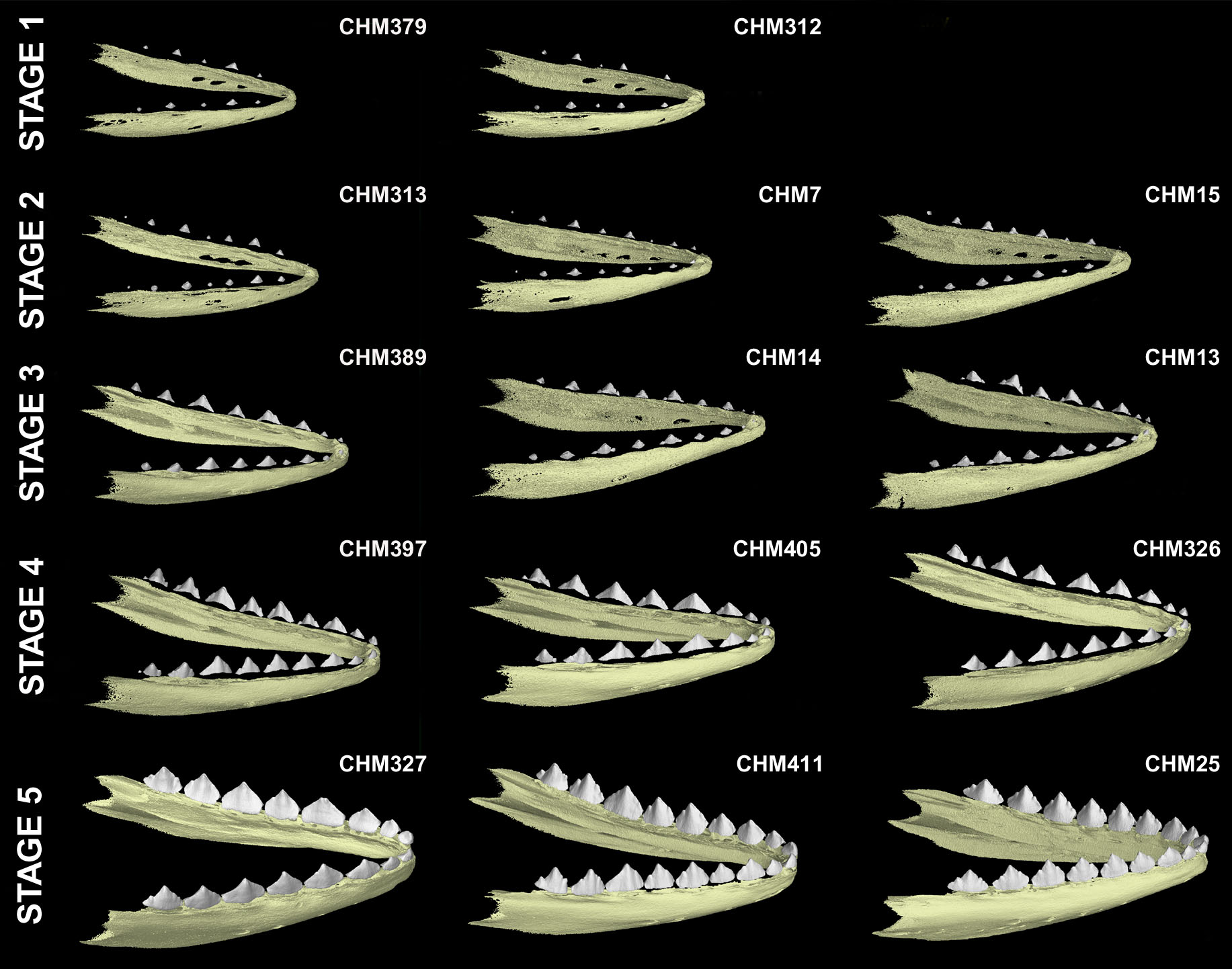

Supplement: Supplementary file 2 — Supplementary Figure S1. [file 41598_2020_78939_MOESM2_ESM.jpg]

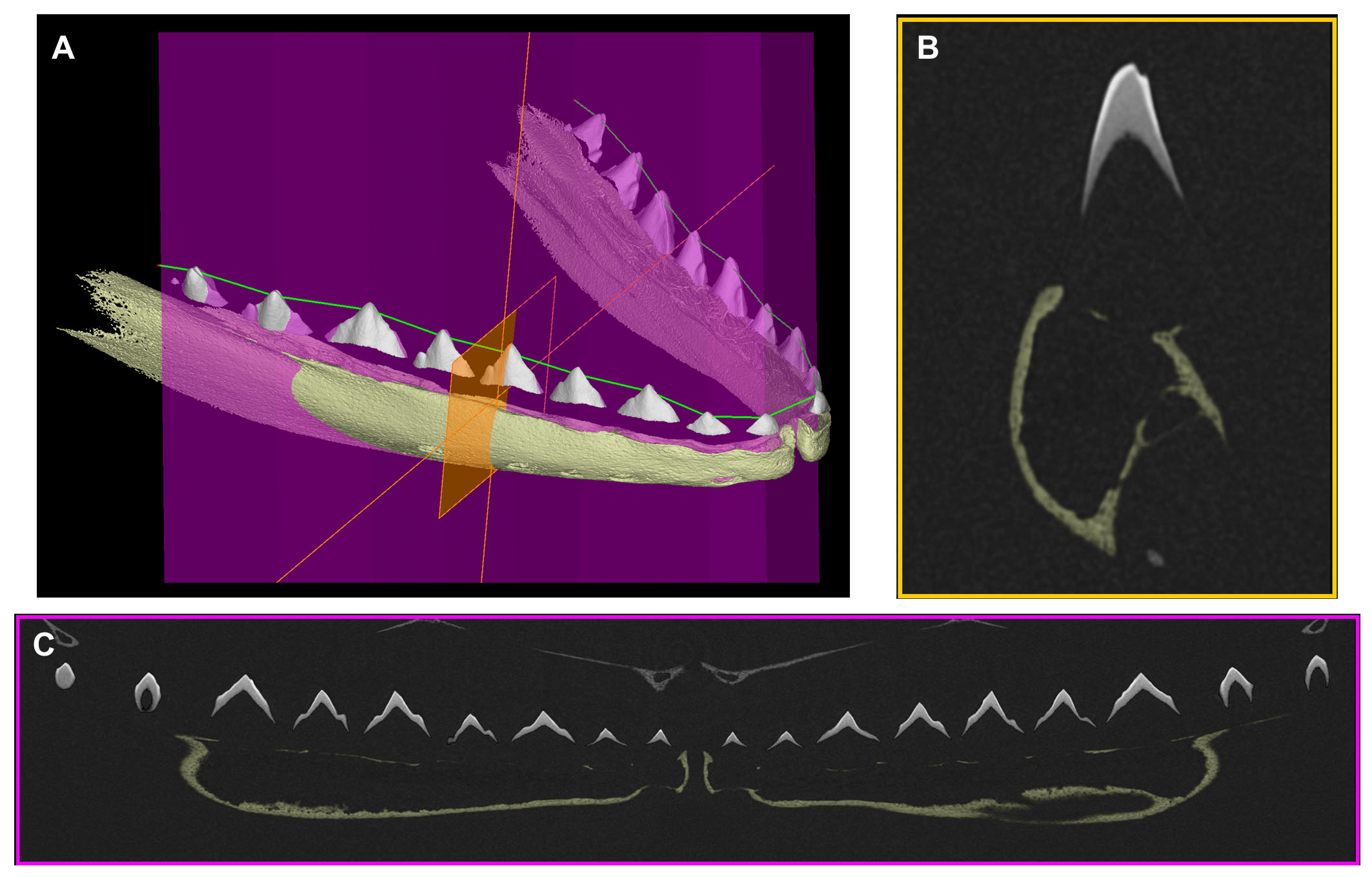

Supplement: Supplementary file 3 — Supplementary Figure S2. [file 41598_2020_78939_MOESM3_ESM.jpg]

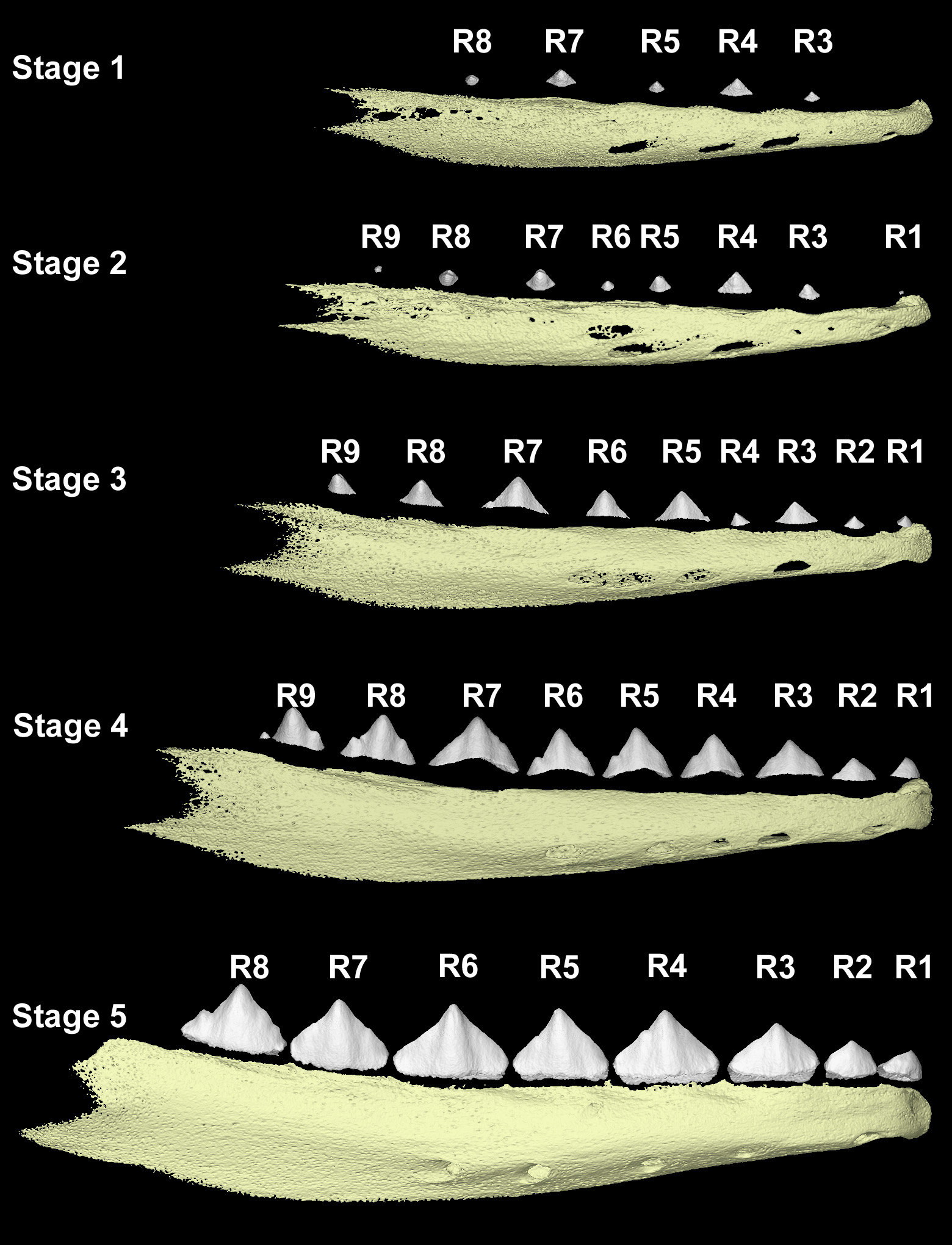

Supplement: Supplementary file 4 — Supplementary Figure S3. [file 41598_2020_78939_MOESM4_ESM.jpg]

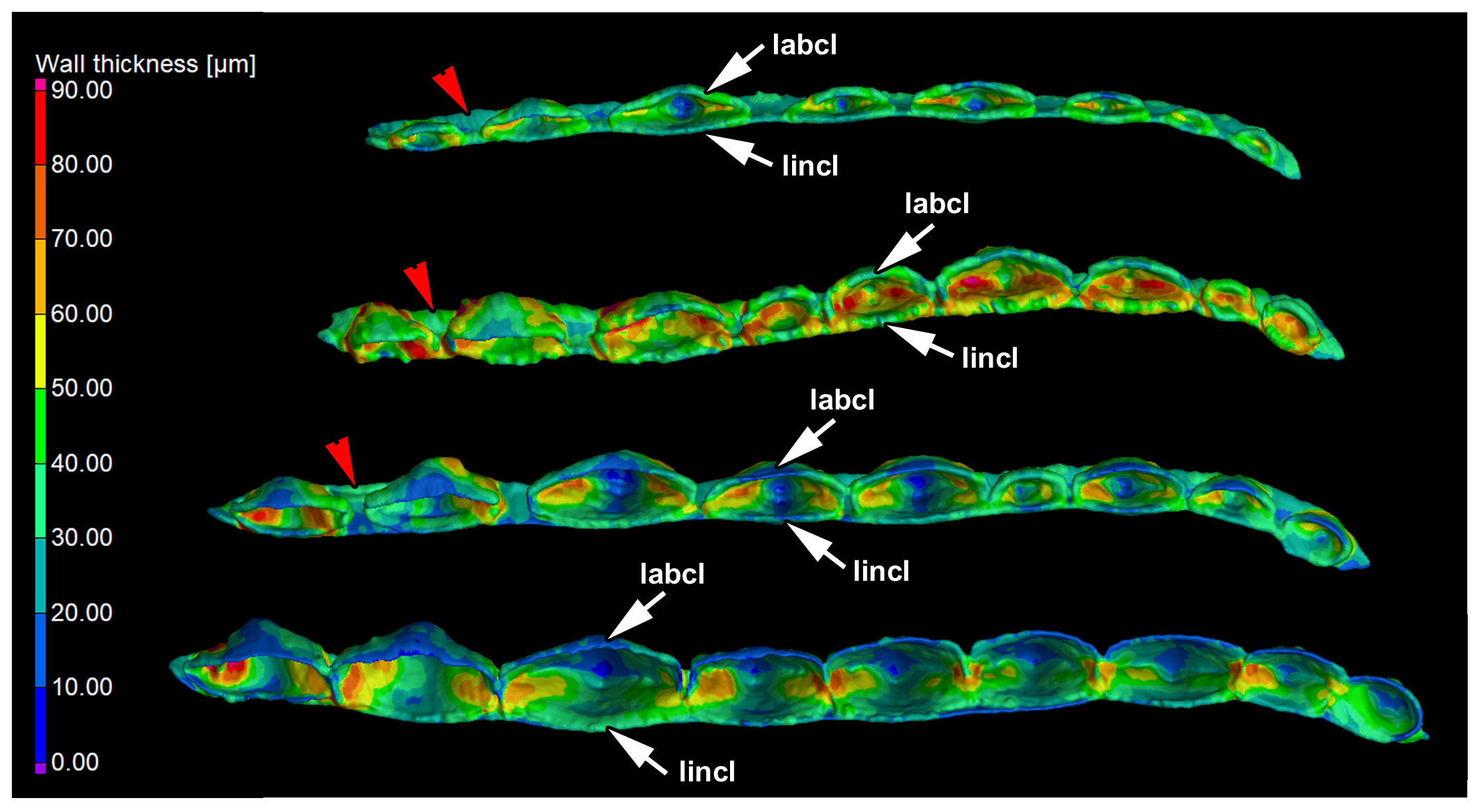

Supplement: Supplementary file 5 — Supplementary Figure S4. [file 41598_2020_78939_MOESM5_ESM.jpg]

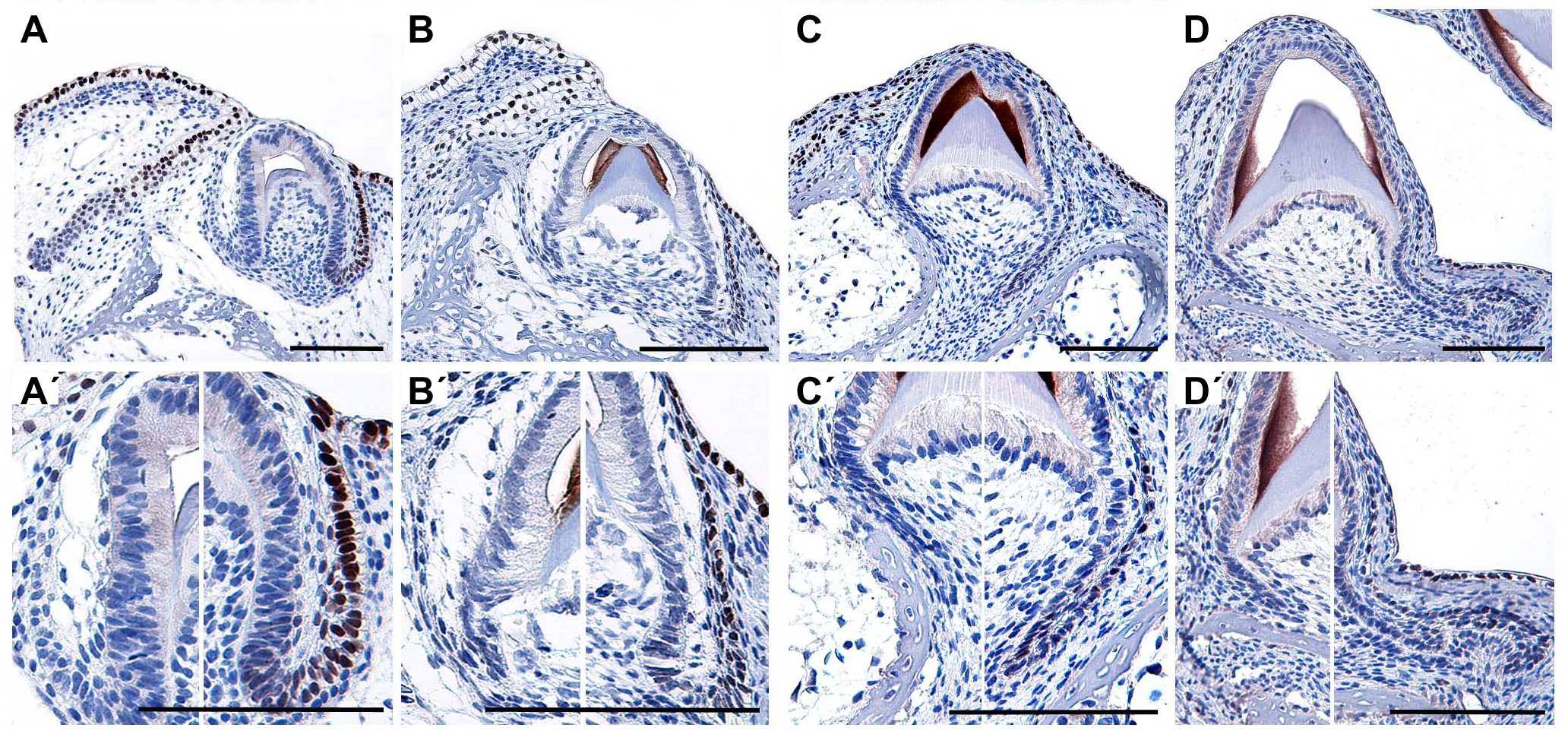

Supplement: Supplementary file 6 — Supplementary Figure S5 [file 41598_2020_78939_MOESM6_ESM.jpg]

# Stage 1

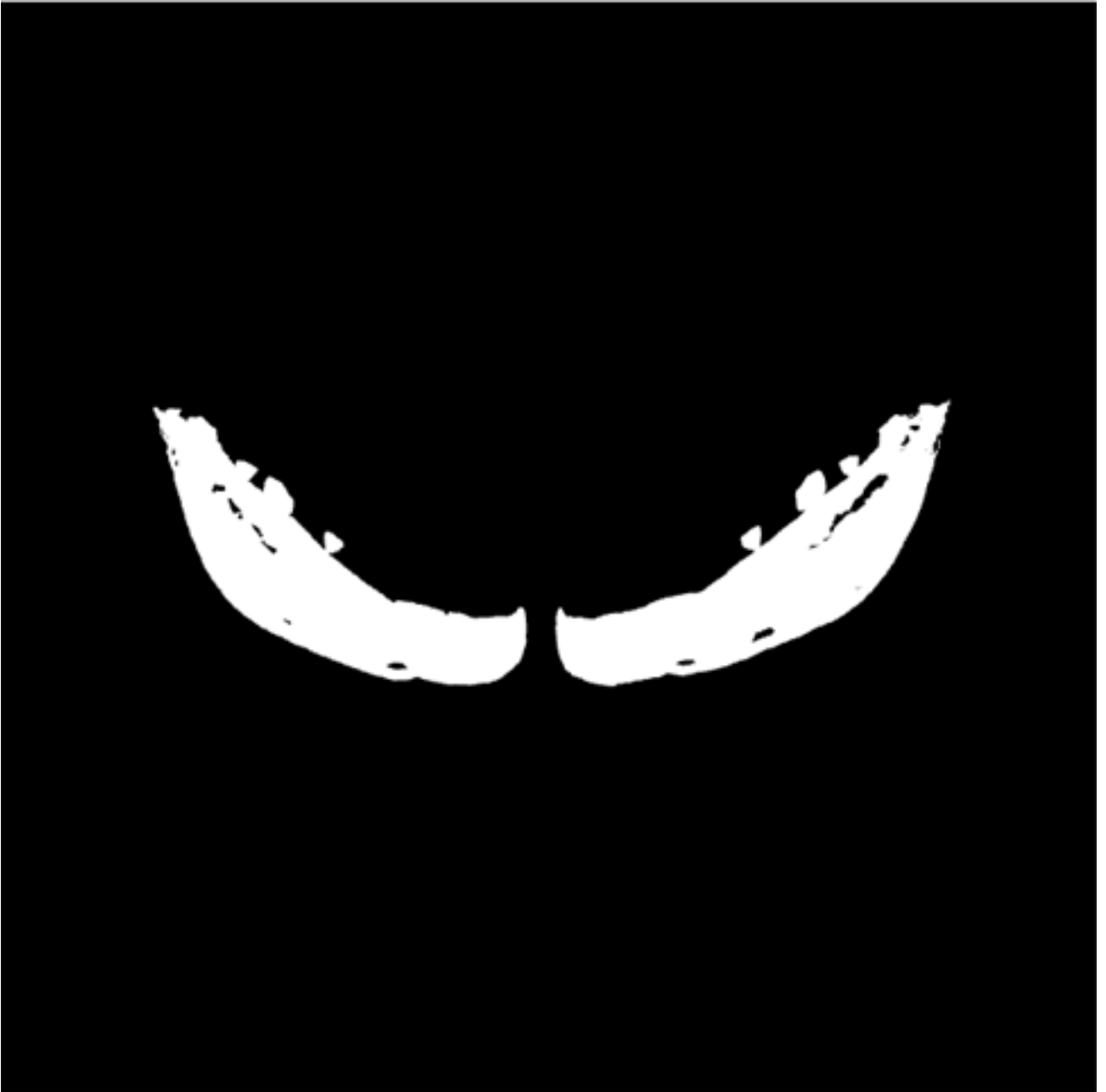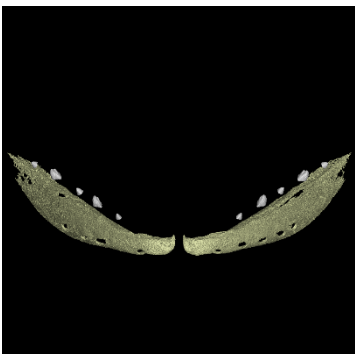

bone + teeth

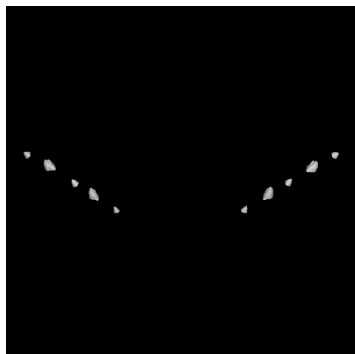

teeth

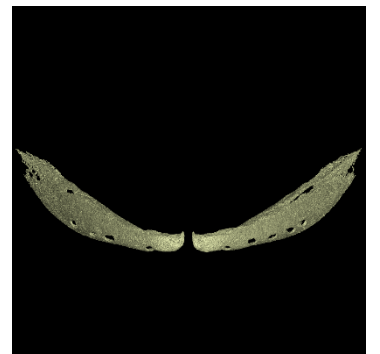

bone

Supplement: Supplementary file 7 — Supplementary Figure S6 [file 41598_2020_78939_MOESM7_ESM.pdf]

# Stage 2

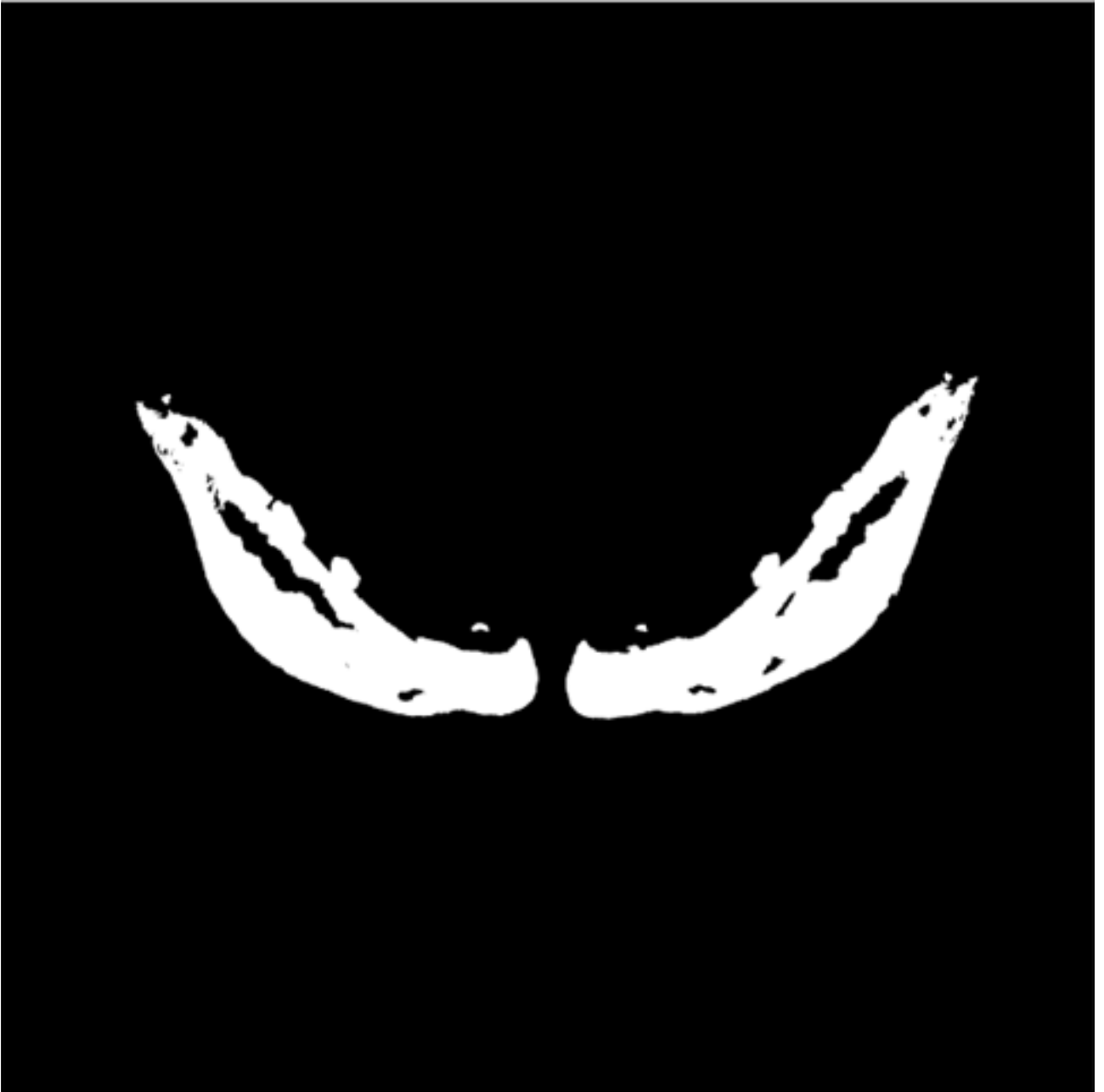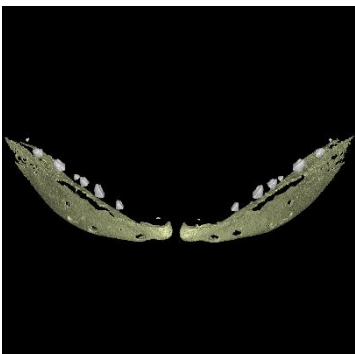

bone + teeth

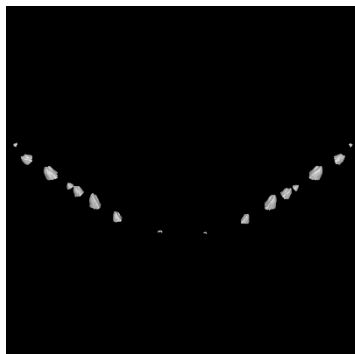

teeth

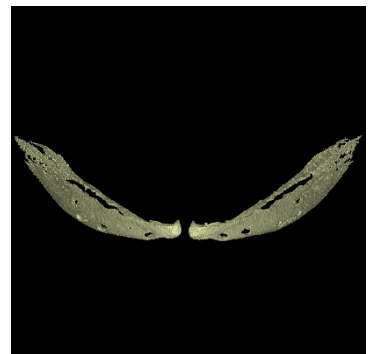

bone

Supplement: Supplementary file 8 — Supplementary Figure S7 [file 41598_2020_78939_MOESM8_ESM.pdf]

# Stage 3

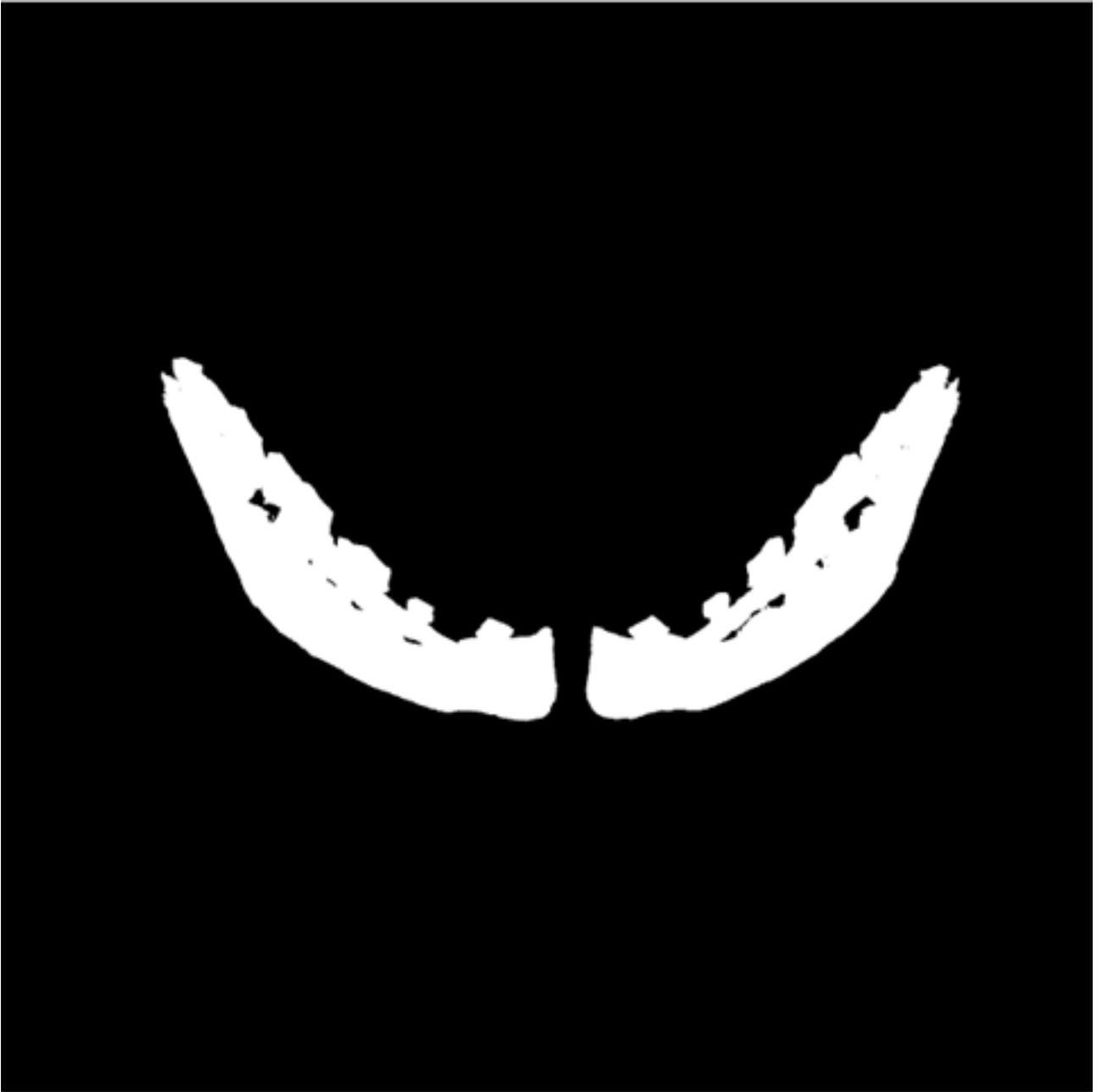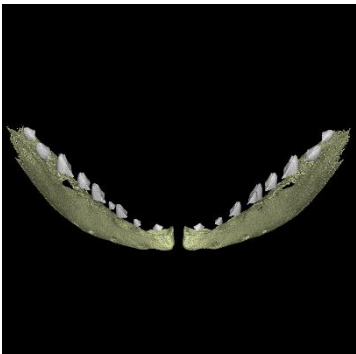

bone + teeth

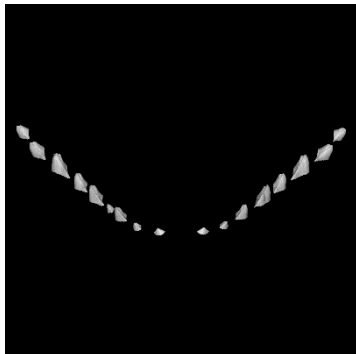

teeth

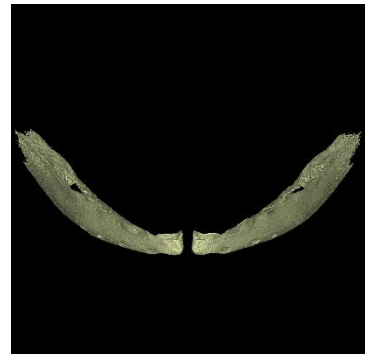

bone

Supplement: Supplementary file 9 — Supplementary Figure S8. [file 41598_2020_78939_MOESM9_ESM.pdf]

# Stage 5

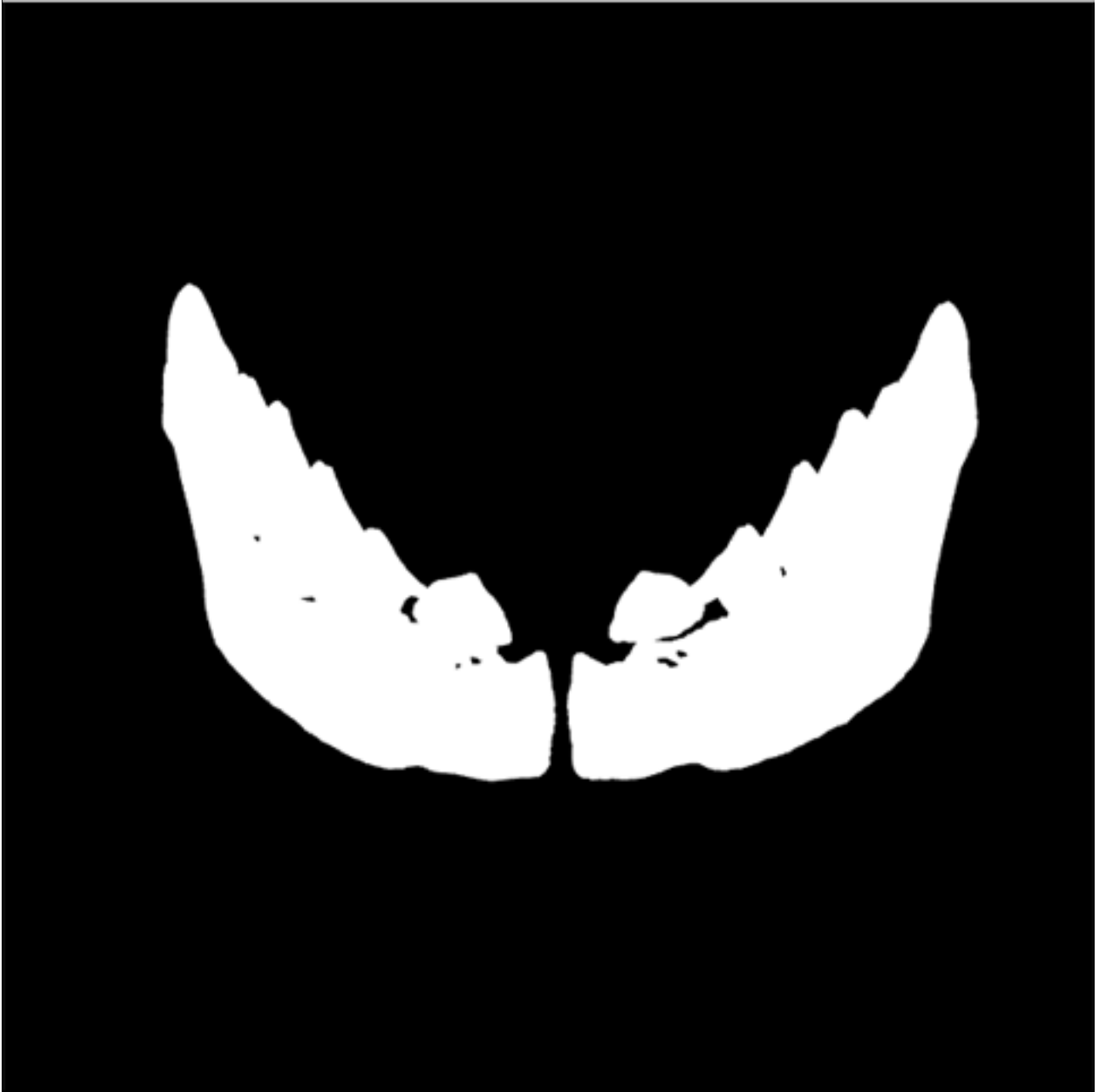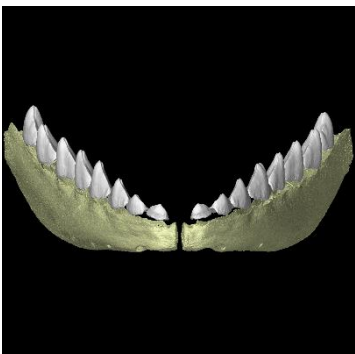

bone + teeth

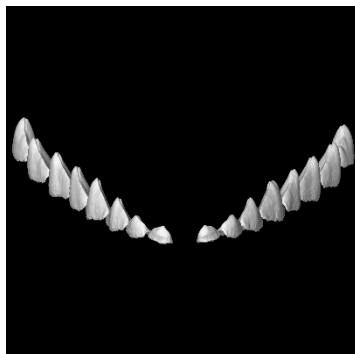

teeth

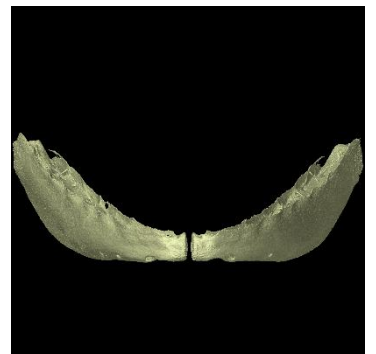

bone

Supplement: Supplementary file 10 — Supplementary Figure S9. [file 41598_2020_78939_MOESM10_ESM.pdf]
